# Bond Behavior of Glass Fiber-Reinforced Polymer (GFRP) Bars Embedded in Concrete: A Review

**DOI:** 10.3390/ma18143367

**Published:** 2025-07-17

**Authors:** Saad Saad, Maria Anna Polak

**Affiliations:** Department of Civil and Environmental Engineering, University of Waterloo, Waterloo, ON N2L 3G1, Canada

**Keywords:** Glass Fiber-Reinforced Polymer (GFRP) Reinforcement, bond–slip, composites, calibration, experimental testing, bond model

## Abstract

Glass Fiber-Reinforced Polymer (GFRP) bars are becoming increasingly common in structural engineering applications due to their superior material properties, mainly their resistance to corrosion due to their metallic nature in comparison to steel reinforcement and their improved durability in alkaline environments compared to CFRP and BFRP reinforcement. However, GFRP bars also suffer from a few limitations. One of the main issues that affects the performance of GFRP reinforcing bars is their bond with concrete, which may differ from the bond between traditional steel bars and concrete. However, despite the wide attention of researchers, there has not been a critical review of the recent research progress on bond behavior between GFRP bars and concrete. The objective of this paper is to provide an overview of the current state of research on bond in GFRP-reinforced concrete in an attempt to systematize the existing scientific knowledge. The study summarizes experimental investigations that directly measure bond strength and investigates the different factors that influence it. Additionally, an overview of the analytical and empirical models used to simulate bond behavior is then presented. The findings indicate the dependence of the bond on several factors that include bar diameter, bar surface, concrete strength, and embedment length. Additionally, it was concluded that both traditional and more recent bond models do not explicitly account for the effect of different factors, which highlights the need for improved bond models that do not require calibration with experimental tests.

## 1. Introduction

Glass Fiber-Reinforced Polymers (GFRP) are increasingly common in structural engineering applications (bridge reinforcements, seismic retrofitting, and concrete strengthening) due to their superior material properties [1]. For example, pultruded GFRP sections have been used as I- and C-sections in structural applications [2]. Additionally, GFRP bars have been used as the main reinforcement in concrete structures exposed to seawater [3]. The feasibility of using GFRP bars in structural applications has been shown in [4,5,6]. The properties that make GFRP bars suitable for such applications include high tensile strength, high strength-to-weight ratio, corrosion resistance, and cost effectiveness over the long term [7]. However, GFRP bars also suffer from several limitations that might hinder their widespread use. These limitations include low ductility, low modulus of elasticity, and low flexural strength [8]. Another issue that can negatively affect the performance of GFRP bars in reinforced concrete structures is the bond slip between the concrete and the GFRP bars [9]. Bond slip refers to the relative displacement that occurs between the reinforcing bar and the surrounding concrete and characterizes the load transfer between the concrete and the rebar through adhesion, friction, and mechanical interlocking. Due to their different material properties and surface textures, the bond behaviour of GFRP bars is different than that of steel bars. While steel bars are manufactured with ribs that enhance mechanical interlock by creating substantial bearing and shearing resistance along the length, GFRP bars usually have smoother surfaces and thus are unlikely to achieve the same level of mechanical interlock. Additionally, the ductility of steel bars allows the ribs to deform under load, which enhances mechanical interlock. On the other hand, the brittleness of GFRP bars limits their ability to form additional interlocks. This reduction in mechanical interlock in GFRP bars can be compensated for by increased friction that results from the use of coatings and other surface treatments. Additionally, while adhesion plays a minor role in the bond behavior of steel rebars, that role is further reduced in GFRP bars since the polymer matrix is largely inert to cement paste, which results in minimal chemical adhesion. Thus, the bond mechanism in steel rebars is mainly dominated by mechanical interlock, while the bond mechanism of GFRP bars is a combination of mechanical interlock and friction.

However, while the bond between concrete and steel has been thoroughly investigated throughout the years, GFRP bars have only recently been adopted in concrete structures, and therefore, their bond–slip behavior has not been fully characterized. Therefore, it is essential to perform a comprehensive review of the bond behavior of GFRP bars embedded in concrete and the factors that influence this bond to ensure a safe and reliable design. Such a review is also relevant to other types of FRP bars, as bond behavior depends on the surface of the FRP bars, and the findings of this review could apply to other types of FRP bars, such as CFRP and BFRP.

**Figure 1:**
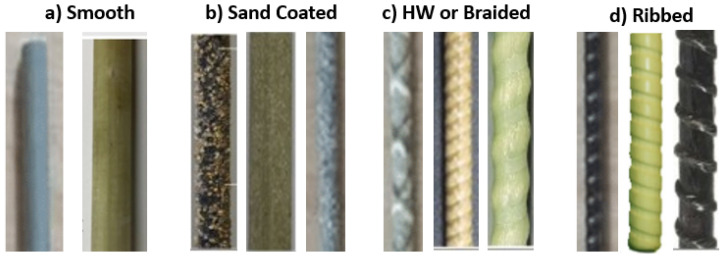
Different types of bar surfaces: (**a**) smooth bars; (**b**) sand-coated bars; (**c**) HW or braided bars; and (**d**) ribbed bars. Based on [24].

**Figure 2:**
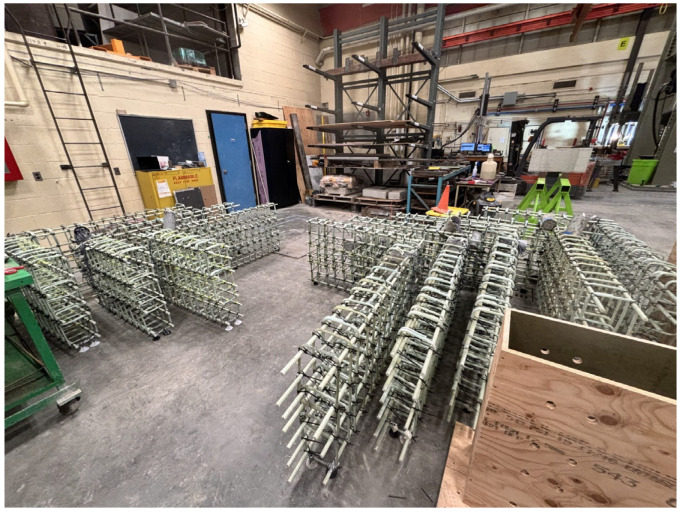
GFRP reinforcement cages for use in concrete corner joints.

The manufacturing process has a significant effect on the bond behavior and other mechanical properties of GFRP bars [10,11,12,13,14,15,16,17,18,19,20,21,22]. In addition, bond strength can be significantly influenced by other factors, such as concrete compressive strength, bar surface, bar size, bar location, and embedment length. Several experimental studies have been conducted to better understand the bond behavior of GFRP bars. For example, Ehsani et al. [23] experimentally investigated the impact of GFRP surface preparation (Figure 1 shows different surface types while Figure 2 shows GFRP bars in application), loading conditions, concrete compressive strength, bar diameter, clear cover distance, and top bar effects on bond strength [23]. Furthermore, several attempts have been made to model bond behavior in GFRP-reinforced concrete empirically and theoretically. The models use experimental data to establish and calibrate relationships between bond strength and various influencing factors.

While individual studies that investigate the bond behavior of GFRP bars, either experimentally or numerically, are available, a comprehensive review is needed to integrate fragmented research and enable the consolidation of dispersed knowledge, which will allow researchers and practitioners to access findings from a wide range of studies. The review would also help identify the key factors influencing the bond behavior of GFRP bars and help evaluate bond modelling accuracy to reveal suitability for practical applications. Furthermore, a systematic review provides a robust evidence base to help in the development and refinement of design codes and guidelines.

The objective of this paper is to provide an overview of the current state of research on bond behavior in GFRP-reinforced concrete. The study summarizes experimental investigations that directly measure bond strength and investigates the different factors that influence it. An overview of the analytical and empirical models used to simulate bond behavior and calibrate them against experimental results is then presented. Additionally, an overview of the expressions used in research and design guidelines to estimate the peak bond strength is also provided. A summary of current knowledge is provided, research gaps are identified, and practical application implications are highlighted. The database was compiled from various scientific publications indexed in leading databases, such as Scopus and Elsevier. The selection criteria for including journals in this review not only emphasized the impact factor and disciplinary relevance but also the rigor of peer review processes to ensure the inclusion of high-quality research.

## 2. Experimental Studies

**Figure 3:**
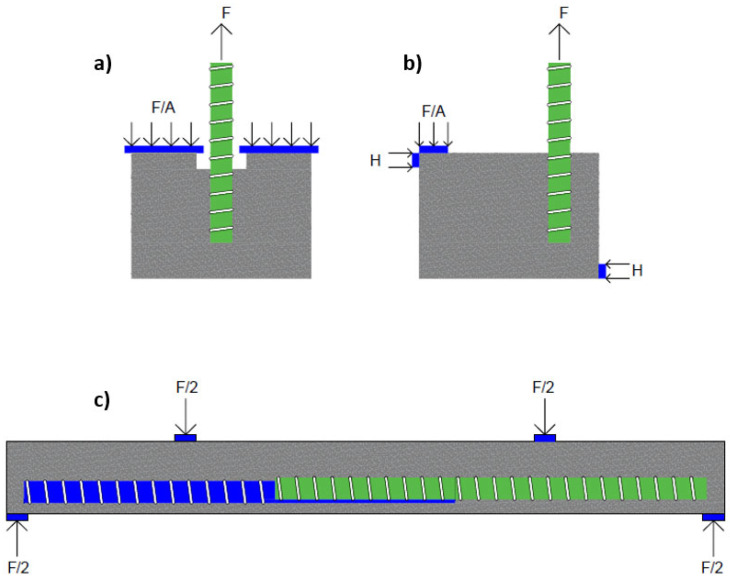
(**a**) Pullout test; (**b**) beam end test; and (**c**) lap splice beam test.

The bond strength of reinforcing bars is typically measured using either pullout tests or beam tests [25,26]. The pullout test involves measuring the force required to directly pull out a bar embedded in the center of a concrete cylinder or prism. This kind of test provides direct measurements of the force magnitude and the corresponding slip, which can then be translated into a bond stress–slip relationship [27]. The bond stress can be calculated from the force as per Equation (1), where τ is the bond stress representing the strength of the bond between the steel and concrete, F is the pullout force, d is the bar diameter, and l is the embedment length. On the other hand, beam tests involve loading a specially designed reinforced concrete beam to bending failure, allowing researchers to observe and quantify how the bars slip relative to the concrete under realistic stress conditions [28,29]. While beam tests provide a more realistic estimate of bond strength, the value obtained from this test is usually a lower bound, while the pullout test provides an upper bound estimate. The reason for this discrepancy is that the main failure mechanism in pullout tests is pullout failure. Splitting of concrete is avoided in pullout tests due to the absence of local bending on the bar, higher thickness of the concrete cover, and the confining action of the reaction plate on the concrete specimen [30]. On the other hand, failure in beam tests may be caused by either pulling out or splitting. However, despite the larger accuracy associated with beam tests, researchers tend to prefer pullout tests due to their simplicity [30,31]. It is noted that there are different types of beam and pullout tests (such as the standard pullout test, the eccentric pullout tests, the direct tension pullout test, the RILEM RC5 Beam test, and the lap splice beam test), with the standard pullout being the preferred test for bond slip in GFRP bars [32]. Furthermore, both pullout and beam end tests are covered in testing standards. Pullout tests are covered in ASTM C900 [33] and EN 12504-3 [34], while beam tests are covered in standards, such as ASTM A944 [35] and IS 2770-1 [36]. Figure 3 depicts a standard pullout test, a beam end test, and a lap splice beam test. As mentioned, bond failure is characterized by either pullout or splitting failure. Pullout failure typically occurs in specimens with short embedment lengths and sufficient confinement, where bond stress is transferred without causing significant damage to the surrounding concrete and is characterized by bar slippage out of the concrete without significant cracking or damage to the surrounding concrete mass. In contrast, splitting failure arises when radial stress exceeds the tensile strength of the concrete, often in cases of longer embedment lengths, larger bar diameters, insufficient cover, or poor confinement and is characterized.

(1) $$\tau =\frac{F}{\left(\pi \cdot d\cdot l\right)}$$

**Figure 4:**
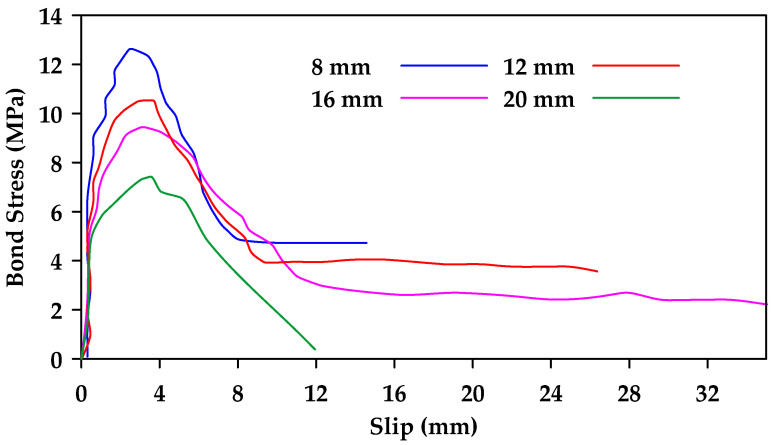
Bond stress–slip relation for helically wrapped GFRP bars with varying diameters in unconfined concrete. Data from [37].

**Figure 5:**
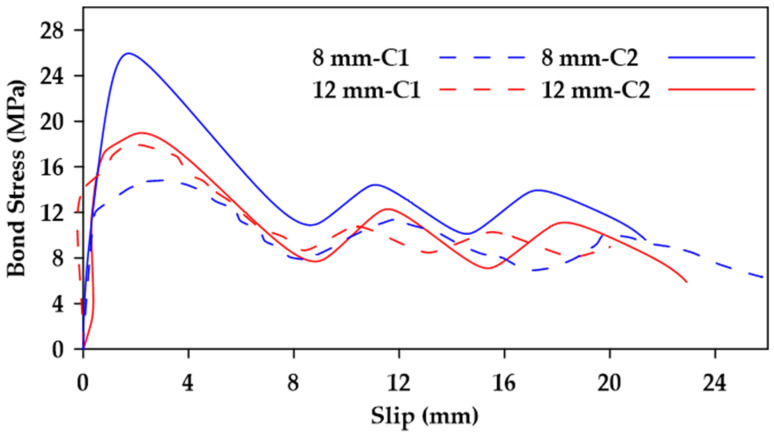
Bond stress–slip relation for 8 and 12 mm ribbed GFRP bars embedded in concrete of different strengths (C1 and C2 (28 and 48 MPa, respectively)). Data from [37].

**Figure 6:**
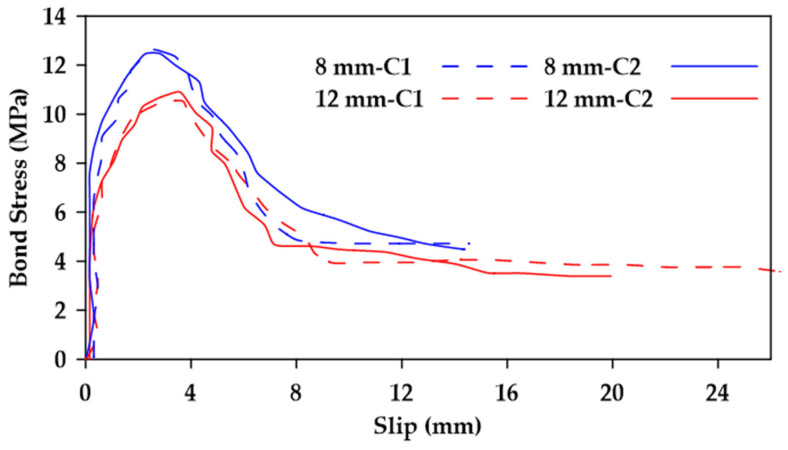
Bond stress–slip relation for 8 and 12 mm non-ribbed GFRP bars embedded in unconfined concrete of different strengths (C1 and C2 (28 and 48 MPa, respectively)). Data from [37].

Gao et al. [37] performed pullout tests on GFRP bars embedded in concrete and reported on the various variables that impinge upon bond behavior [37]. The variables considered included the surface characteristics of the bars, the bar diameter, the concrete strength, as well as the confinement effect of stirrups. Four nominal diameters 8, 12, 16, and 20 mm) and three types of external surfaces were studied (helical wrapping, helical wrapping with sand coating, and ribbed). Additionally, two different concrete strengths were investigated (28 MPa and 40 MPa). Furthermore, specimens with and without 8 mm stirrups spaced at 40 mm were tested. The embedment length used was 5 d, where *d* represents the bar diameter. The findings indicate that the bond strength decreases as the bar diameter increases as highlighted in Figure 4. Moreover, it was observed that increases in concrete strength led to an increase in bond strength in specimens where bond failure occurs at the surfaces of both the bar and the concrete (i.e., ribbed bars) as shown in Figure 5. This effect is more prominent in bars with smaller diameters. On the other hand, bond strength in specimens where failure occurred at the surface of the bar only (i.e., non-ribbed or smooth bars) was not affected by concrete strength (Figure 6).

**Figure 7:**
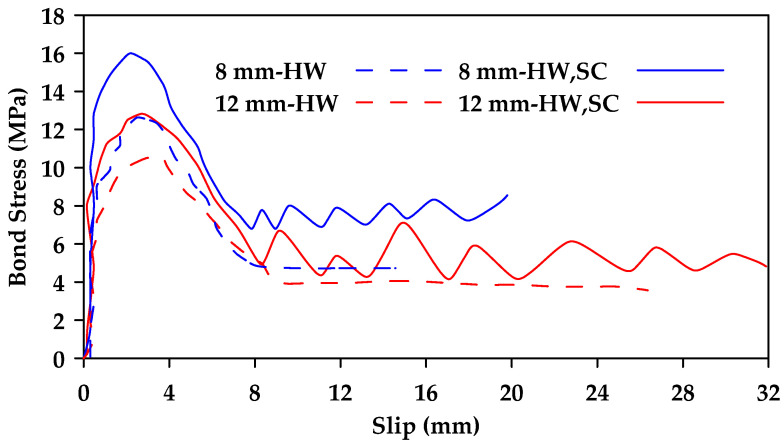
Comparison between bond stress–slip relationships, sand-coated (HW, SC) and non-sand-coated helically wrapped (HW) 8 and 12 mm GFRP bars embedded in concrete (C1). Data from [37].

**Figure 8:**
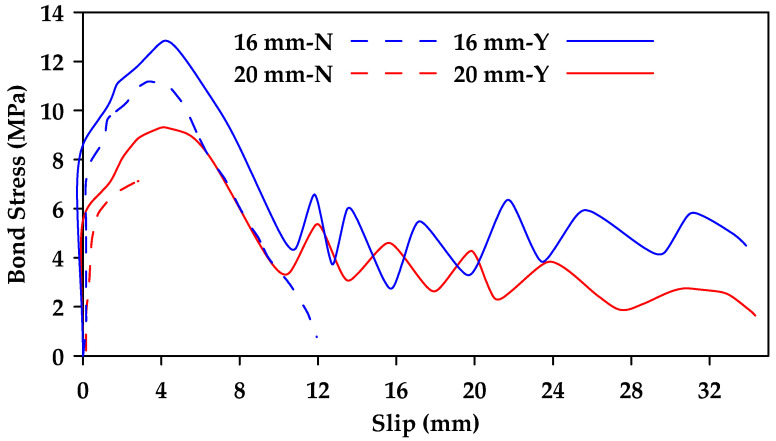
Comparison between bond stress–slip relationships for sand-coated helically wrapped 16 and 20 mm GFRP bars embedded in concrete (C1) with and without confining stirrups (Y represents confinement and N represents no confinement). Data from [37].

**Figure 9:**
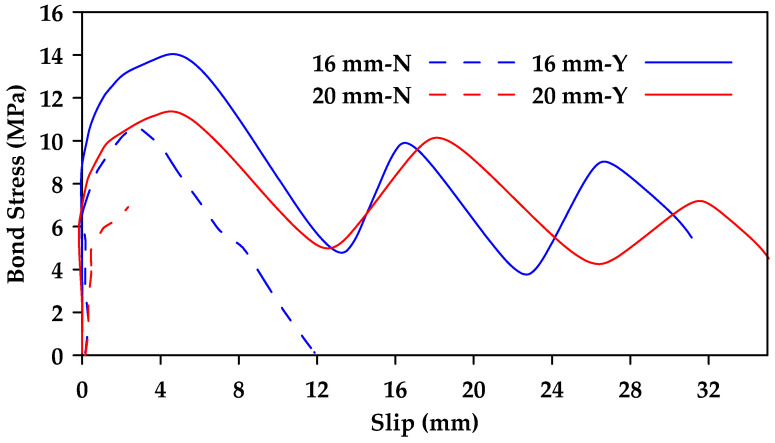
Comparison between bond stress–slip relationships for ribbed 16 and 20 mm GFRP bars embedded in concrete (C1) with and without confining stirrups (Y represents confinement and N represents no confinement). Data from [37].

Additionally, it was concluded that surface characteristics have a significant effect on bond behavior, with ribbed bars exhibiting a higher bond strength than helical wrapped bars and sand-coated helical bars [37]. Failure in ribbed bars occurred due to the crushing of concrete and the shearing of ribs. In contrast, the concrete surface remained intact when testing helical wrapped bars and sand-coated helical bars, with failure resulting from the detachment of the fiber spirals and resin layer in the helical wrapped bars and from the detachment of sand grains in the sand-coated bars. Furthermore, the sand-coated helical bars exhibited greater bond strength than traditional helical bars (Figure 7). It was also found that the presence of stirrups can change the failure mode of the specimen from concrete splitting failure to pullout failure, correspondingly increasing the bond strength of the GFRP bars and improving overall bond behavior (Figure 8 and Figure 9).

**Figure 10:**
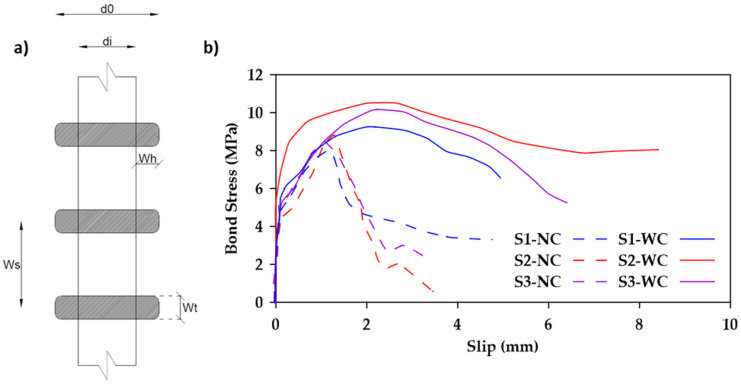
(**a**) Geometric properties of ribbed bars and (**b**) Comparison between bond stress–slip relationships for GFRP bars embedded in concrete with (WC) and without confining stirrups (NC). Data from [38].

In another study, Rather et al. [38] investigated the bond behavior of 24 GFRP-reinforced concrete specimens [38]. The bond strength increased by up to 15% when the surface geometric properties of the GFRP bars were altered (i.e., winding span, winding thickness, and winding height as shown in Figure 10). This occurs because of improved mechanical interlocks and friction mechanisms. It was also observed that the bond strength decreased as the embedment length of the GFRP rebars increased. This reduction may be attributed to the nonlinear stress distribution and the inconsistent reduction of frictional forces over the embedment length of the bar. Furthermore, the use of confining stirrups led to an increase of about 10–18% in bond strength. It was also noted that all specimens containing confining reinforcement exhibited a relatively ductile pullout failure, as shown in Figure 10b, where the notations S1, S2, and S3 represent different bar surfaces having different combinations of winding span, winding thickness, and winding height.

**Figure 11:**
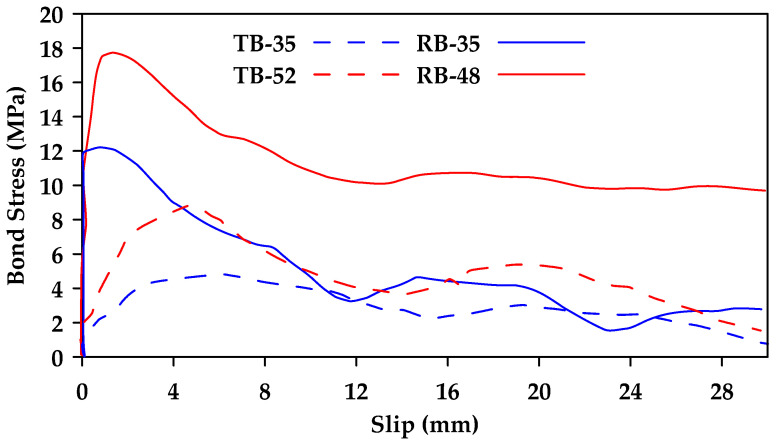
Bond stress–slip relationships obtained for threaded (TB) and ribbed (RB) bars in unconfined concretes of different strengths. Note that the number in the notation represents the concrete strength in MPa. Data from [39].

The pullout local bond stress–slip response of GFRP bars was also investigated in [39]. The test parameters included the concrete cover, the splice length, the effect of confinement provided by stirrups, and the concrete compressive strength. The findings indicated that the presence of confining reinforcement increases the bond strength significantly for both ribbed bars, which experienced splitting failure, and thread-wrapped bars, which experienced pullout failure. Furthermore, it was concluded that increasing the width of the concrete cover has a less pronounced effect on the bond strength in GFRP bars than in steel bars of equal diameter. Increasing concrete strength led to an increase in bond strength in both types of GFRP bars, with ribbed bars having higher bond strength than threaded bars (Figure 11).

**Figure 12:**
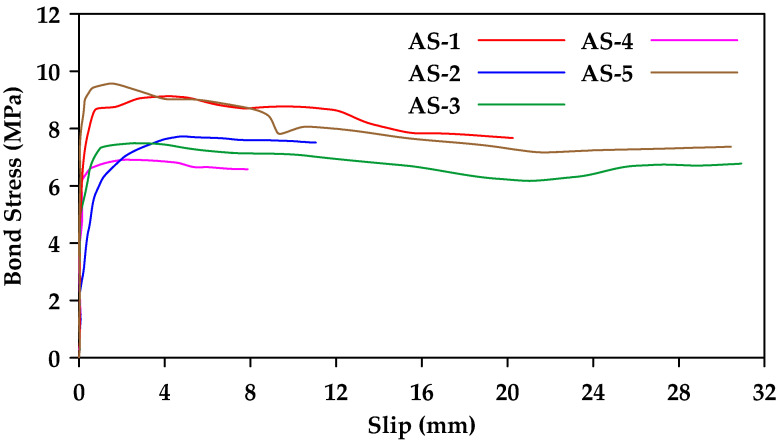
Bond stress–slip relationships obtained for No. 4 GFRP bars (5 samples). Data from [40].

In a different study, Gooranorimi et al. [40] performed pullout tests on GFRP bars with a nominal diameter (d) of 12.7 mm [40]. The bars in consideration were manufactured of helically wrapped fiber and coated with fine sand. Five different samples with an embedment length of 16d were investigated. All the samples experienced pullout failure with an average bond strength of 8.53 MPa and a standard deviation of 0.81 MPa (Figure 12).

**Figure 13:**
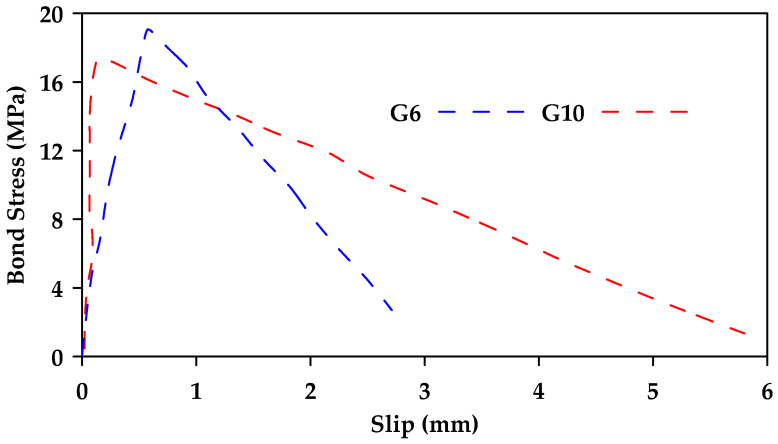
Bond stress–slip relationships obtained for GFRP bars of diameter 6.4 and 9.5 mm (G6 and G10, respectively). Data from [41].

Ahmed et al. [41] conducted pullout testing of 40 FRP (Glass and Carbon) and steel bars [41]. The study included four different diameters of sand-coated GFRP bars (6.4, 9.5, 12.7, and 15.9 mm). The bars were embedded in concrete with a compressive strength of 38 MPa and an embedment length equal to 5d. All tested samples (both steel and GFRP) failed due to bar pullout. Furthermore, it was observed that the bond strength decreased with an increase in bar diameter. Figure 13 shows the obtained bond stress–slip relationships for GFRP bars of diameter 6.4 and 9.5 mm (G6 and G10, respectively). The average bond strength for GFRP samples with a diameter of 6.4, 9.5, 12.7, and 15.9 mm was 20, 17.5, 14, and 13.5 MPa, respectively.

**Figure 14:**
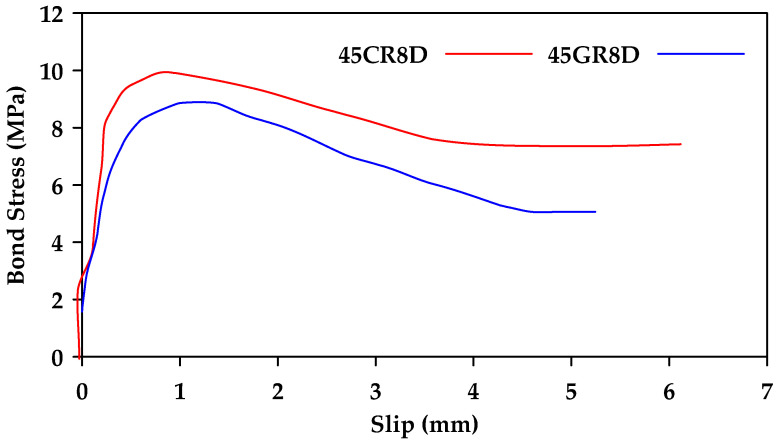
Bond stress–slip relationships for round GFRP (45Gr8D) and CFRP bars (45Cr8D) embedded in 45 MPa concrete. Data from [42].

Achillides and Pilakoutas [42] performed direct pullout tests on 130 specimens of concrete reinforced with different types of bars (Glass, Carbon, Aramid FRP and steel) [42]. The main factors considered were the concrete strength, the diameter of the bar, the shape of the bar (round or square), and the embedment length. Figure 14 shows the obtained bond stress–slip relationships for round GFRP and CFRP bars embedded in 45 MPa concrete. The two bar types behave similarly, with the CFRP bars having a slightly higher bond strength than the GFRP bars. On the other hand, a significant difference was observed between the failure modes of steel and GFRP bars in concrete with strength greater than 30 MPa. When steel bars were tested, shear cracks developed between the bars and the concrete before pullout failure occurred. This implies that the bond strength of steel bars is highly dependent on the strength of concrete. On the other hand, the bond strength of GFRP bars was found to be independent of the concrete strength when the concrete has a strength greater than 30 MPa. For concrete strength less than 30 MPa, failure occurs in the concrete matrix for both steel and GFRP, and thus, the bond strength of both types of bars is dependent on the concrete strength. Additionally, the bond strength recorded in square GFRP bars was found to be about 25% higher than that of round GFRP bars. Another finding was that the increase in bar diameter leads to a decrease in bond strength of GFRP bars.

**Figure 15:**
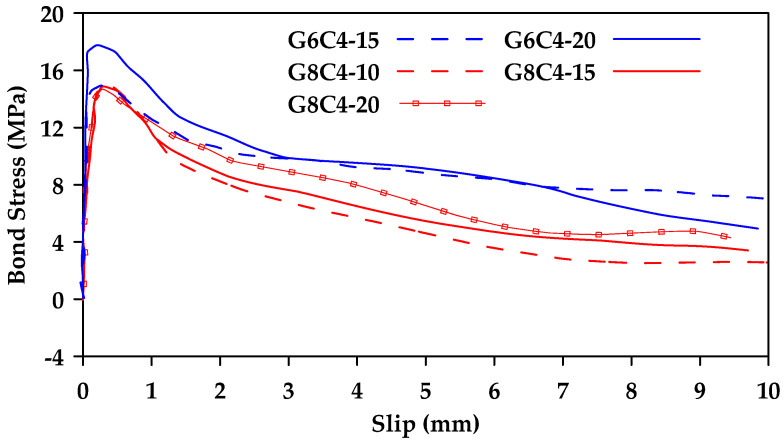
Bond stress–slip relationships for 6 and 8 mm GFRP bars (G6 and G8, respectively) embedded in C4 concrete (62 MPa) with varying concrete covers (10, 15, and 20 mm). Data from [43].

Veljkovic et al. [43] compared the performance of GFRP bars and steel bars subjected to pullout tests [43]. Two different types of GFRP bars (wrapped and ribbed) were tested. The effects of the concrete material properties and the concrete cover were also considered. The results indicated that sanded and spirally wrapped GFRP bars have brittle bond behavior, while ribbed GFRP bars behave in a manner similar to steel bars. Furthermore, ribbed GFRP bars have higher bar slips for maximum value of bond stress, compared to sanded and wrapped bars, but slightly smaller values compared to steel. It was also found that the bond strength of ribbed GFRP bars is dependent on the concrete mechanical strength. The material properties of concrete with average compressive strength between 25 and 40 MPa do not significantly affect the bond strength. On the other hand, the bond strength is significantly enhanced when GFRP bars are embedded in concrete with an average compressive strength within the range of 40–65 MPa. Additionally, it was observed that increasing the concrete cover can lead to a decrease in bond strength of ribbed GFRP bars embedded in concrete with low compressive strength and can cause a change in the failure mode from direct concrete splitting to crushing of concrete. On the other hand, this effect is reversed as concrete strength increases (as shown in Figure 15). Furthermore, in cases of concrete with high compressive strength, the effect of concrete cover decreases as the bar diameter increases (Figure 15). For optimal benefits, it was recommended that ribbed GFRP bars be used in conjunction with mid-strength concrete (~55 MPa) and a low concrete cover to produce a high bond strength and delay cover cracking.

**Figure 16:**
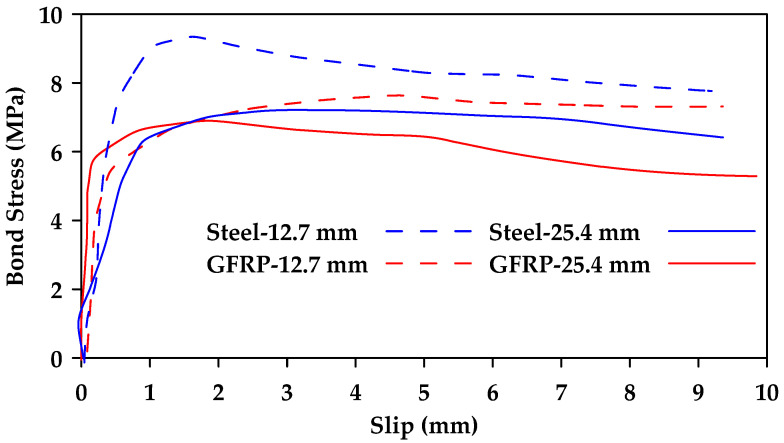
Load–slip relationships obtained from beam tests performed on GFRP and steel bars of different diameters. Data from [44].

Benmokrane and Tighiouart [44] studied the bond strength of GFRP and steel bars by conducting beam tests on 20 concrete beams reinforced with four nominal diameters from 12.7 to 25.4 mm [44]. The findings confirmed that the bond strength of GFRP bars decreases with an increase in bar diameter. The reported bond strength of GFRP reinforcing bars was lower than that of steel reinforcing bars, depending on reinforcing bar diameter, as shown in Figure 16.

In another study, Tastani and Pantazopoulou [32] investigated the local bond mechanics of glass fiber-reinforced GFRP bars in normal-strength concrete by conducting 30 direct pullout tests on GFRP bars [32]. The study considered the impact of the bar surface and bar diameter. The two types of GFRP bars investigated were Aslan100 and Fiberglass CPP rebars, with the main difference between the two being in their surface pattern. The Aslan bars were sand coated with helical lengthwise indentations, while the CPP bars were sand coated only. The diameter considered for each type of bar were 12.7, 15.88, and 19.05 mm. The results for the CPP bars showed that the bond strength decreases with an increase in bar diameter, as has been observed from previous tests. However, the results for the Aslan100 bars indicated that the bar diameter has minimal effect on bond strength in this type of bar surface. The authors attributed this to the fact that Aslan100 bars of different sizes had varying surface indentation, which altered bond conditions between bars of different diameters.

**Figure 17:**
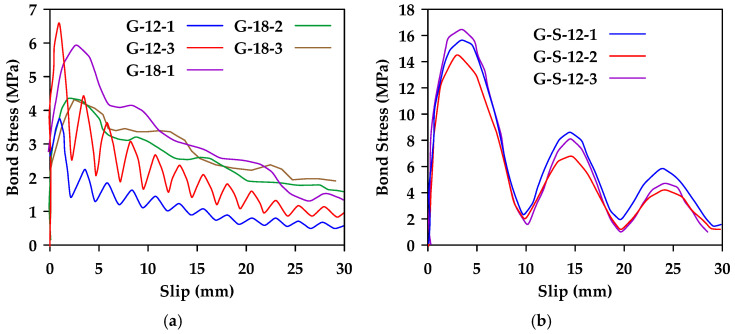
Bond stress–slip relationships for 12 and 18 mm GFRP bars with (**a**) shallow or (**b**) no ribs (S represents shallow rib). Note that the last number represents sample number. Data from [45].

Chen et al. [45] conducted pullout tests on three types of bars (steel bars, glass fiber-reinforced polymer (GFRP) bars, and basalt fiber-reinforced polymer (BFRP) bars) with different surface conditions (smooth and ribbed for steel bars and smooth, shallow ribbed, and deep ribbed for BFRP bars and GFRP bars) [45]. Two bar diameters (12 and 18 mm) were considered. The authors observed that all GFRP bars with no or shallow ribs with a diameter of 12 mm exhibited pullout failure. The 18 mm smooth GFRP bars also exhibited pullout failure. On the other hand, 18 mm GFRP bars with shallow and deep ridges and the 12 mm GFRP bars with deep ridges exhibited premature splitting failure. Figure 17 shows the results obtained for smooth and shallow ribbed GFRP bars of different diameters. It was also concluded in this study that rib height has a great influence on bond behavior, and bond strength is increased with larger rib height.

**Figure 18:**
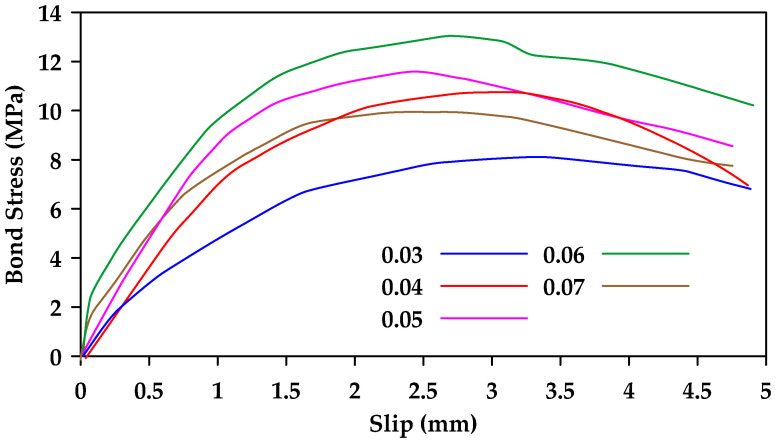
Bond–slip relationships for 12 mm GFRP bars with different rib height (each color represents a different height as a percentage of the rebar diameter). Data from [46].

Hao et al. [46] performed pullout tests on 30 types of GFRP bars with different rib geometries to investigate the effect of rib height and rib spacing [46]. When rib spacing was kept constant, the bond strength of ribbed rebars with a rib height equal to 6% of the rebar diameter was superior (Figure 18). On the other hand, when rib height was constant, the bond strength of ribbed rebars with a rib spacing equal to the rebar diameter was superior. Thus, the optimal combination was having a rib spacing equal to the rebar diameter and a rib height equal to 6% of that value.

In addition to the aforementioned studies, both pullout and bending tests were performed on GFRP bars and steel bars embedded in concrete in [47]. Furthermore, Wu et al. [48] performed lap splice beam tests to determine the effect of stirrups spacing on bond behavior of GFRP bars [48]. As expected, it was determined that decreasing bond spacing leads to an increase in bond strength, particularly in bars with a smaller diameter. Abbas et al. [49] also investigated the effect of lap splicing and stirrups spacing on GFRP bar bond [49]. Zhao et al. [50] compared the bond behavior of GFRP bars and the bond behavior of CFRP and steel bars through pullout testing [50]. Vint [51] concluded that the surface profile of GFRP bars can significantly influence the post-peak phase of the bond stress–slip curve [51]. Solyom and Balázs [52] investigated the bond behavior of GFRP bars with different surface configurations. The configurations studied included helically wrapped, sand coated, helically wrapped and sand coated, indented, and ribbed surfaces. The findings indicated significant differences in the bond strength obtained between bars of different surfaces. Moreover, significant differences were also observed between bars of the same surface category (e.g., varying sand fineness within the sand-coated surface category) [52]. Other experimental studies on the bond behavior of GFRP bars embedded in concrete and the parameters that affect it include [53,54,55,56,57,58,59,60,61,62,63,64,65].

**Figure 19:**
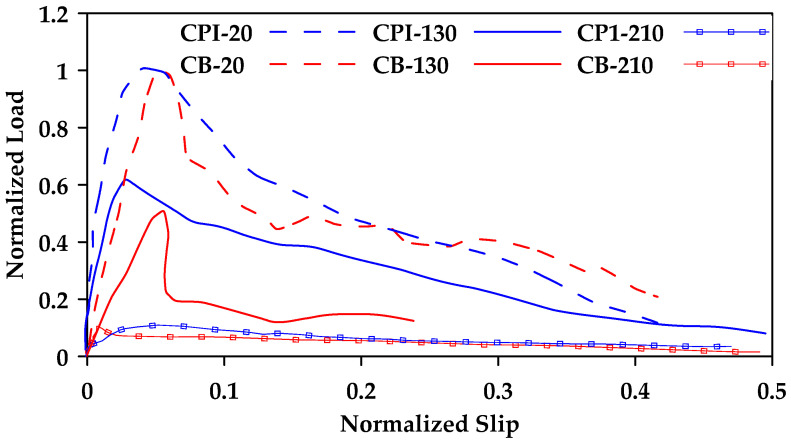
Effect of temperature on bond strength slip in CB and CPI GFRP bars. Data from [66].

**Figure 20:**
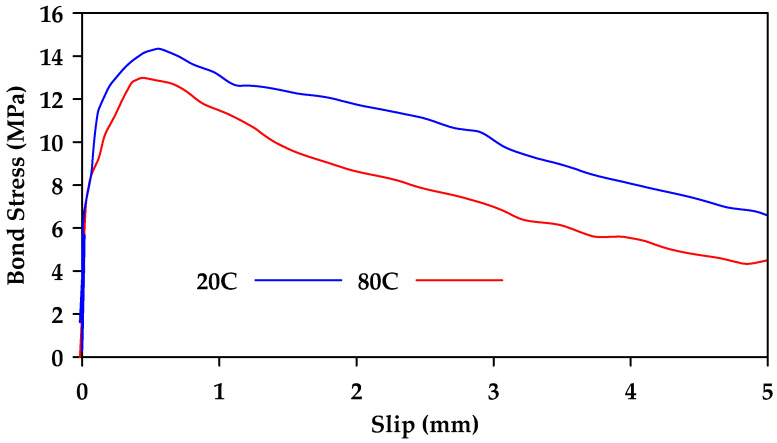
Load–slip relationships obtained for the 8 mm GFRP bars at temperatures of 20 and 80 °C. Data from [68].

The effect of elevated temperatures on the bond strength of GFRP bars embedded in concrete has also been studied. In [66], it was determined that regardless of surface texture, elevated temperatures significantly influence the bond strength. Initially, the strength loss observed in GFRP bars due to increased temperatures is similar to the bond loss observed in steel bars. However, as temperatures increased beyond 100 °C, a significant loss in bond strength was observed with a reduction of about 90% in strength at a temperature of 200 °C as shown in Figure 19 where the notation CB refers to GFRP bars with molded deformations on the surface, similar to ordinary deformed steel rebars, with a smooth surface between deformations and the notation CPI refers to GFRP bars that contain wraps of a wide braid of fibers on the surface. Similar findings were also reported in [67], where it was found that the bond strength measured at 20 °C was reduced by 80–90% when the temperature increased to 220 °C. In another study, Masmoudi et al. [68] subjected a total of 80 specimens to four different temperatures (20, 40, 60, and 80 °C) for four and eight months [68]. Two different bar diameters were investigated (8 and 16 mm). Minimal effect was observed as temperatures increased from 20 °C to 60 °C. However, as temperatures increased to 80 °C, the maximum pullout load decreased by up to 15% (Figure 20). Hajiloo and Green [69] also performed pullout tests on sand-coated and ribbed GFRP bars for a temperature range of 25 °C–360 °C [69]. It was determined that the bond strength significantly decreased as temperature increased, with a maximum of 17% retained strength at temperatures above 200 °C regardless of bar surface. Mousavi et al. [70] investigated the effectiveness of longer embedment lengths on bond degradation due to temperature [70]. It was determined that the increase in embedment length led to improved pullout capacity under elevated temperatures [70].

Ellis et al. [71] observed that 27% of the original bond strength was retained once GFRP bars cooled down to room temperature after being exposed to temperatures of 400 °C [71]. Hamad et al. [72] found that the bond strength of 10 mm GFRP bars was reduced by 82% when exposed to temperatures of 325 °C [72]. Özkal et al. [73] reported that steel bars retained 60% of their bond to concrete at an elevated temperature of 600 °F, while GFRP bars only retained 34% of their bond strength at the same temperature [73]. It is noted, however, that the tested specimens were of different diameters (12 mm for the steel bars and 9 mm for the GFRP bars). Solymon et al. [74] also performed pullout tests on indented GFRP bars embedded in concrete and exposed to temperature ranges of 20 °C to 300 °C [74]. All specimens failed due to bar pullout regardless of temperature, and it was concluded that bond strength decreases with temperature, with a reduction of 90% in bond strength observed at a temperature of 300 °C. In addition, it was concluded that the reduction of bond strength with temperature is also affected by the reduction of concrete strength and elasticity with temperature. Furthermore, the effect of effect of bond degradation at elevated temperature on structural performance of GFRP-reinforced concrete structures was also studied in [75,76,77,78].

Ruiz Emparanza et al. [79] investigated the bond behavior between GFRP bars and concrete in marine environments [79]. In this study, pullout tests were performed on GFRP bars subjected to different temperatures (23,  40, and 60 °C) and exposed to seawater for 60 and 120 days. The results show that the bond behavior is independent of the duration of exposure and bond deterioration was not observed throughout the testing period. The resistance of GFRP bars to aggressive environments was also confirmed by pullout tests conducted in [80]. In those tests, the GFRP bars were exposed to twelve different harsh environments. Alves et al. [81] investigated the effect of freeze-thaw cycles and fatigue loading on the bond behavior of GFRP bars [81]. It was determined that fatigue load can reduce the bond strength by about 50% while freeze-thaw cycles can improve the bond to concrete by about 40%. Abed and El Mesalami [82] concluded that the bond between GFRP bars and concrete is minimally affected by exposure to direct sunlight and seawater [82]. The resistance of the bond between GFRP bars and concrete to aggressive environments (such as alkaline solutions, acid solutions, and salt solutions) was also confirmed in [80,83,84,85,86,87,88,89,90,91]. Furthermore, Robert and Benmokrane [92] concluded that aging has a minimal effect on the durability of the GFRP bar–concrete interface [92].

Several attempts have been made to investigate the bond stress–slip relationships of GFRP bars to different types of concrete. Saleh et al. [93] performed hinged beam tests on 28 specimens of GFRP bars embedded in high-strength concrete [93]. Most of the tested specimens failed due to the pullout of the GFRP bar. Additionally, it was determined that bond strength reduces with an increase in embedment length and bar diameter. The bond characteristics of GFRP bars in high-strength concrete were also investigated in [94]. Hossain et al. [95] performed pullout tests on GFRP bars embedded in ultra-high-strength concrete [95]. Hu et al. [96] investigated the bond behavior of sand-coated GFRP bars embedded in different types of concrete (Ultra-High-Performance Concrete (UHPC) and High-Performance Concrete (HPC)) [96]. The findings indicated that GFRP bars embedded in UHPC showed an increase of about 72% and 164% in bond strengths when compared against GFRP bars embedded in HPC and normal concrete, respectively. Furthermore, it was found in [97] that the reduction rate of bond strength of GFRP bars with increasing bar diameter and embedment length was reduced in high-strength concrete when compared against GFRP bars embedded in normal concrete. Luo et al. [98] investigated the effect of bar diameter on the bond of ribbed GFRP bars embedded in UHPC and determined that the bond initially increases with an increase in bar diameter up to a certain limit (18 mm), above, which the bond strength decreases with increased diameter [98]. Lu et al. [99] found that sand-coated bars exhibited better bond behavior than ribbed bars when embedded in high-performance concrete [99]. Tong et al. [100] also investigated the bond behavior of GFRP bars in UHPC [100]. In another study, it was determined that the use of seawater sea-sand ultra-high-strength concrete instead of freshwater river-sand concrete has minimal effect on the bond strength of GFRP bars [101]. Other studies have been performed to determine the bond behavior of GFRP bars in self-compacting concrete [102,103,104,105]. Furthermore, Romanazzi et al. [106] determined that the bond strength of sand-coated GFRP bars increased by 55% when embedded in geopolymer concrete rather than ordinary Portland cement [106]. Other studies on bond behavior of GFRP bars embedded in geopolymer concrete were conducted in [107,108]. Doostmohamadi et al. [109] investigated the effect of different types of concrete (lightweight concrete and lightweight fiber-reinforced concrete) on the bond of GFRP bars [109]. The bond behavior of GFRP bars in coral concrete has also been investigated in [110,111,112].

**Figure 21:**
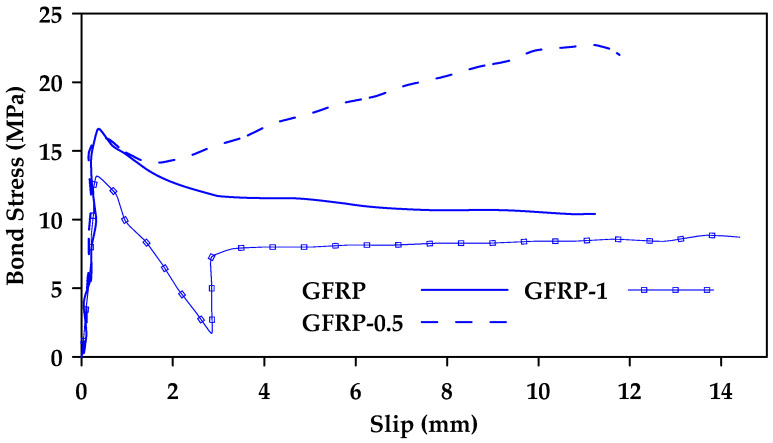
Comparison between bond strength of smooth GFRP bars (notation A) with 0.5% steel fibers and 1% steel fibers. Data from [113].

Kim et al. [113] investigated the effect of using structural fibers on the bond between GFRP bars and concrete by performing pullout tests on cubic concrete specimens reinforced with GFRP bars [113]. The main differences between the specimens were in the fiber type (steel, PVA, and PP), the fiber volume fraction, and the GFRP bar surface (sand coated and helically wrapped). It was found that the use of structural fibers leads to an increase in bond strength as well as a delay in the pullout failure of the bars. Moreover, the addition of structural fibers changed the failure modes for GFRP bars. The authors recommended the use of different volume fractions and different types of structural fibers for different types of bars: 0.5% steel fibers for sand-coated GFRP rebar (Figure 21) and 1% PVA fibers for helically wrapped GFRP rebar. Haung et al. [114] investigated the bond behavior of GFRP bars embedded in fiber-reinforced concrete containing three different types of artificial fibers (carbon, aramid and polypropylene) [114]. It was concluded that the bond behavior of GFRP bars was significantly improved when embedded in concrete containing fibers as opposed to ordinary concrete. Won et al. [115] studied the bond behavior of GFRP bars embedded in high-strength concrete containing varying amounts of steel and synthetic fibers [115]. The obtained bond strength between the FRP reinforcing bar and the concrete increased by 5–70% as the volume fraction of fiber increased [115].

Ashrafi et al. [116] investigated the use of carbon fiber mat anchorage to improve the bond behavior of GFRP bars [116]. The bond strength increased by up to 20% when the anchorage system was used efficiently (i.e., in specimens where confinement strength was sufficient). Rahimi et al. [117] found that using steel anchors can improve the bond strength of GFRP bars to concrete by up to 60% depending on the concrete compressive strength, bar diameter, and geometry of the steel anchor [117]. Shakiba et al. [118] found that the shape of the anchor head is found to be a key parameter in the bond performance of anchored GFRP bars [118]. Islam et al. [119] determined that headed-end GFRP bars showed improved bond strength in comparison with straight-end GFRP bars [119].

## 3. Analytical Models

Several analytical models that capture bond stress–slip relationships between concrete and FRP bars have been proposed. One such model was proposed in a study conducted by Malvar [120]. In this study, a large array of experimental pullout tests was conducted on GFRP bars. The authors proposed an analytical model for the bond–slip–stress relationship based on their experimental findings. The model, expressed in terms of the bond slip (s) and the bond stress (τ), can be expressed as shown in Equation (2a–c).

(2a) $$\tau =\tau_{1}\frac{F\left(\frac{s}{s_{1}}\right)+(G-1){\left(\frac{s}{s_{1}}\right)}^{2}}{1+\left(F-2\right)\left(\frac{s}{s_{1}}\right)+G{\left(\frac{s}{s_{1}}\right)}^{2}}$$

(2b) $$s_{1}=D+E\sigma_{r}$$

(2c) $$\frac{\tau_{1}}{f_{t}}=A+B(1-e^{-C\frac{\sigma_{r}}{f_{t}}})$$

The empirical constants A,  B,  C,  D,  E, and F are a function of the bar type under consideration. $f_{t}$ is the tensile strength of concrete, $\tau_{1}$ is the peak bond stress, $s_{1}$ is the slip corresponding to the peak bond stress, and $\sigma_{r}$ is the confining axisymmetric radial pressure. It is noted that this study neglected the effect of bar diameter. Another limiting factor is the difficulty of determining the value of $\sigma_{r}$ for members subjected to bending. Furthermore, Malvar’s Model was assessed as being unreliable for modelling the ascending branch of the bond stress–slip relation curve [121].

**Figure 22:**
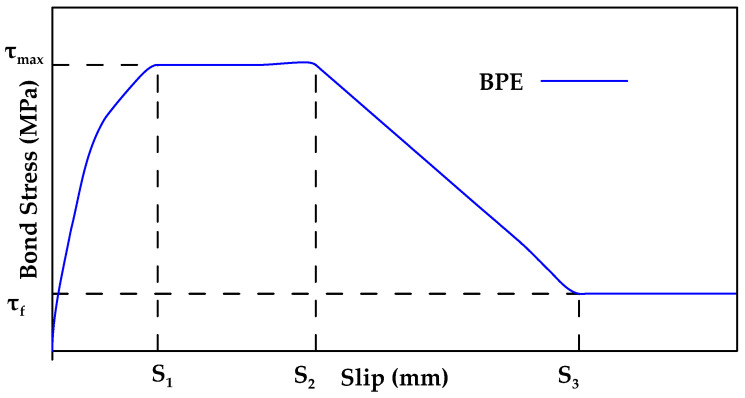
Schematic representation of BPE model. Data from [122].

The BPE model is another model used to describe the bond behavior of FRP bars to concrete [122]. The BPE model comprises an ascending branch, two regions of constant bond stress, and a descending branch as shown in Figure 22. Initially, bond stress increases until the peak bond stress value $\left(\tau_{max},s_{1}\right)$ is reached. This ascending branch corresponds to the chemical adhesion between the bar and concrete, as well as the bearing force. After this point, the bond stress value remains constant while the slip value increases from $s_{1}$ to $s_{2}$. Once point $s_{2}$ is reached, cracks (or even crushing) start to form in the concrete and the bearing force due to mechanical interlocking diminishes, causing the bond stress to decrease linearly until it reaches point $\left(\tau_{3},s_{3}\right)$. Afterwards, the bond stress remains constant due to friction with an increase in slip until failure. In this stage, significant cracking will occur in the concrete [122]. The BPE model can be described mathematically as shown in Equation (3a–d).

(3a) $$\tau =\tau_{max}{\left(\frac{s}{s_{1}}\right)}^{\alpha }\mathrm{for}\;0\leq s\leq s_{1}$$

(3b) $$\tau =\tau_{max}\mathrm{for}\;s_{1}<s\leq s_{2}$$

(3c) $$\tau =\tau_{max}-(\tau_{max}-\tau_{f})(\frac{s-s_{2}}{s_{3}-s_{2}})\mathrm{for}\;s_{2}<s\leq s_{3}$$

(3d) $$\tau =\tau_{f}=\beta \tau_{max}\mathrm{for}\;s_{3}<s$$

In Equation (3a–d), the parameters ($\tau_{max},\;{\;s}_{1},\;{\;s}_{2},\;{\;s}_{3},\;\;\alpha ,$ and β) can be determined using experimental calibration. While the BPE model was designed for steel bars, it has been calibrated for use in FRP bars by [123]. However, even though there was some correspondence between the model and experimental results, several inaccuracies were also identified, particularly in the descending branch. Furthermore, the results obtained were scattered and did not account for bar diameter.

**Figure 23:**
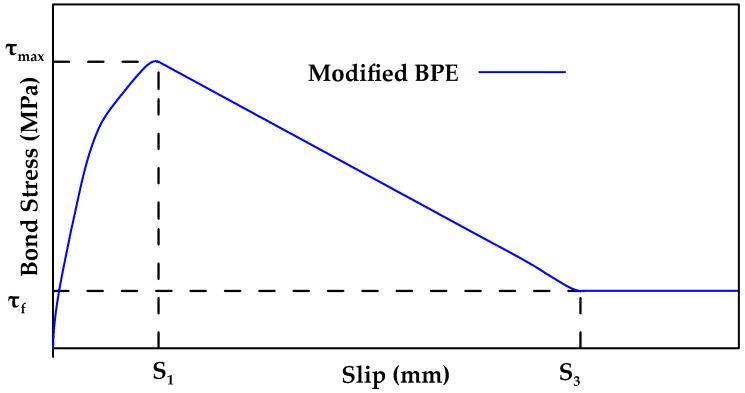
Schematic representation of the modified BPE model. Data from [124].

Cosenza et al. [124] proposed a modification to the BPE model [124]. In the modified BPE (mBPE) model (Figure 23), the equations for the ascending branch are identical to the corresponding equations in the BPE model and only the descending branch equations are altered. The modified BPE (mBPE) model can be described mathematically as shown in Equation (4a–c). In the modified BPE model, only three parameters need to be estimated or calibrated: α, p, and β.

(4a) $$\tau =\tau_{max}{\left(\frac{s}{s_{1}}\right)}^{\alpha }\;\mathrm{for}\;0\leq s\leq s_{1}$$

(4b) $$\tau =\tau_{max}(1-p(\frac{s}{s_{1}}-1))\;\mathrm{for}\;s_{1}<s\leq s_{3}$$

(4c) $$\tau =\tau_{f}=\beta \tau_{max}\;\mathrm{for}\;s_{3}<s$$

Cosenza et al. [125] also proposed a modified ascending branch of the bond–slip–stress relationship [125]. The modified ascending branch provides an initial slope equal to infinity and thus accounts for the physical process of adhesion more accurately. The new proposed model is referred to as the CMR model can be expressed as shown in Equation (5). The parameter φ is an empirical constant determined based on the curve fitting of test data. Note that despite the attempts made to improve the analytical models, none of the models described up to this point consider the bar diameter or surface, which are a major factor in the bond–slip–stress relationship.

(5) $$\tau =\tau_{max}{\left(1-e^{\left(-\frac{s}{s_{1}}\right)}\right)}^{\varphi }\mathrm{for}\;0\leq s\leq s_{1}$$

An alternative modification for the ascending branch was proposed in [53]. In this alternative modification, the bond–slip–stress relationship can be expressed as shown in Equation (6). It should be noted that both the CMR model and the model proposed by [53] are not suitable for design at ultimate limit states as they only account for the ascending branch.

(6) $$\tau =\tau_{max}{\left(1-e^{\left(4s\right)}\right)}^{0.5}\;\mathrm{for}\;0\leq s\leq s_{1}$$

**Figure 24:**
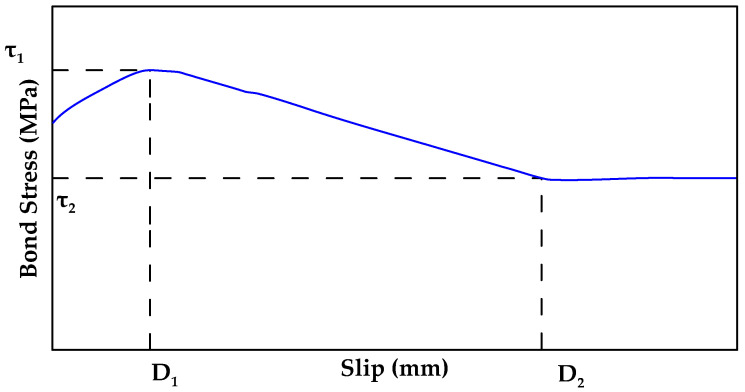
Model proposed by [40].

Another full model was proposed in [40]. The proposed model consists of three stages and five parameters as shown in Figure 24. In this model, τ represents the bond stress and (D) represents the corresponding slip at any point. In the first stage, the bond stress exponentially increases to the peak value of $\tau_{1}$ at the slip level of $D_{1}$ as per Equation (7) (0<a<1 is the exponential parameter). After the bond stress reaches the maximum value of $\tau_{1}$, it linearly decreases to $\tau_{2}$ where the slip reaches $D_{2}$ as per Equation (8).

(7) $$\tau =\tau_{1}{\left(\frac{D}{D_{1}}\right)}^{\alpha }\;\mathrm{for}\;0\leq D\leq D_{1}$$

(8) $$\tau =\left(\frac{\tau_{2}-\tau_{1}}{D_{2}-D_{1}}\right)\left(D_{2}-D_{1}\right)+\tau_{1}\;\mathrm{for}\;D_{1}<D\leq D_{2}$$

Finally, in the last stage, the bond stress remains constant ($\tau =\tau_{2}$). It is noted that the model still requires calibration based on experimental results. For example, Gooranorimi et al. [40] proposed the following values for the model parameters for a 12.7 mm helically wrapped, sand-coated GFRP bar: $D_{1}=1.524\;mm,$ $D_{2}=20.32\;mm$, α=0.17, $\tau_{1}=8.95\;MPa$, and $\tau_{2}=6.93\;MPa$ [40]. This model also does not explicitly account for factors such as bar type, size and coating influence the bond–slip–stress relationship.

Rezazadeh et al. [126] compared two damage-based approaches for assessing the damage evolution of the GFRP bar–concrete bond [126]. One approach was based on the secant modulus model for assessing GFRP bond behavior as developed in [127] while the other approach was based on the exponential damage model developed with the aim of accounting for the interface deterioration of the GFRP–concrete bond. Both approaches involve defining a scalar damage evolution variable D. The value D evolves from 0 at damage initiation to 1 at failure. In both models, a linear elastic zone, in which no damage occurs (D=0), is defined. In this zone, the bond stress increases linearly with the increase in slip. Once the maximum bond stress value $\left(\tau_{b}\right)$ is reached at a slip value equal to $\delta_{b}$, the bond–slip–stress relation is now considered to be in the damaged zone. The two approaches can be quantified as shown in Equation (9a–c).

(9a) $$\tau_{s}=\tau_{e}\mathrm{for}\;\tau_{e}\leq \tau_{b}\to D=0$$

(9b) $$\tau_{s}=\left(1-D\right)\cdot \tau_{e}\mathrm{for}\;\tau_{e}>\tau_{b}$$

(9c) $$\tau_{e}=k_{be}\cdot \delta$$

**Figure 25:**
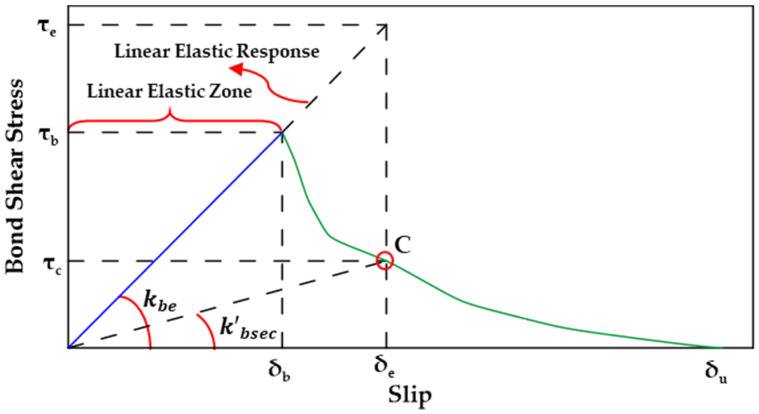
Schematic representation of the secant modulus-based damage model proposed by [126].

In Equation (9a–c), $\tau_{e}$ is the bond stress component predicted by the elastic bond stress–slip relationship without damage and $k_{be}$ is the elastic bond stiffness. The value of $k_{be}$ can be determined as $\frac{\tau_{b}}{\delta_{b}}$. The main difference between the two approaches is in the expression used to define the scalar damage variable D. In the secant modulus-based damage model, the value of D can be determined in accordance with Equation (10a,b). The secant bond stiffnes ${k^{\prime }}_{b\;sec}$ should be determined based on calibration with experimental results. Figure 25 shows a schematic representation of the model.

(10a) $$D=0\mathrm{for}\;\delta \leq \delta_{b}$$

(10b) $$D=1-(\frac{{k^{\prime }}_{b\;sec}}{k_{be}})\mathrm{for}\;\delta >\delta_{b}$$

**Figure 26:**
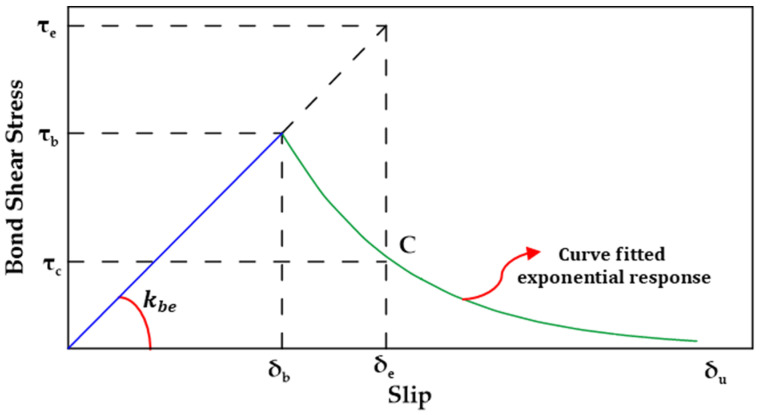
Schematic representation of the exponential damage model proposed by [126].

In the exponential damage approach, the bond shear stress–slip relationship between GFRP bars and concrete in the post-peak phase is described by an exponential function. This function quantifies the damage (D) in terms of the slip (δ) as shown in Equation (11a,b), where $\delta_{u}$ is the slip corresponding to ultimate failure and α is a parameter determined from the fitting of the known bond shear stress–slip curves. Figure 26 shows a schematic representation of the exponential damage approach.

(11a) $$D=0\;\mathrm{for}\;\delta \leq \delta_{b}$$

(11b) $$D=1-(\frac{\delta }{\delta_{b}})(1-\frac{1-e^{\left(-\alpha \left(\frac{\delta -\delta_{b}}{\delta_{u}-\delta_{b}}\right)\right)}}{1-e^{-\alpha }})\;\mathrm{for}\;\delta >\delta_{b}$$

**Figure 27:**
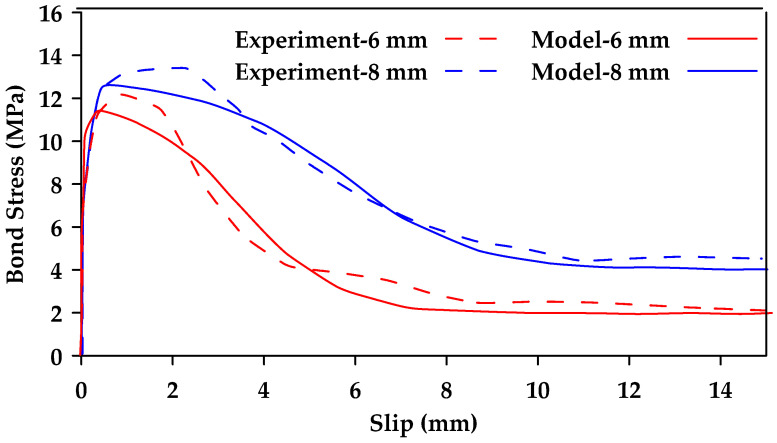
Bond–slip–stress relationship for ribbed steel bars obtained from experimental results and from the proposed model by [128].

**Figure 28:**
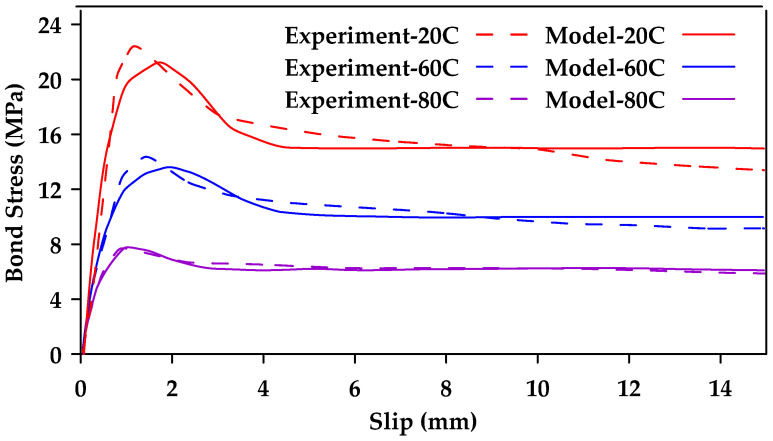
Bond–slip–stress relationships for 10 mm GFRP bars at different temperatures obtained from experimental results and from the proposed model by [128].

**Figure 29:**
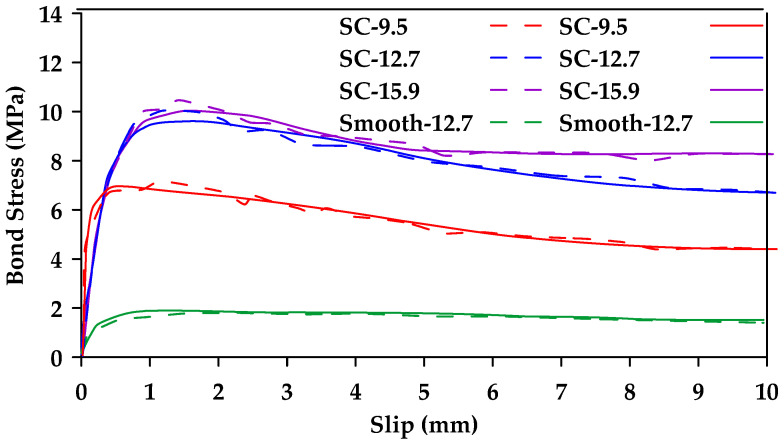
Bond–slip–stress relationships for GFRP bars of different diameters obtained from experimental results and from the proposed model by [128]. SC refers to sand-coated bars.

Furthermore, Biscaia and Carmo [128] proposed a single-function bond–slip model used to simulate the pullout and the detachment process of an embedded rebar from a parent material [128]. The model can be calibrated based on the bar type and the type of parent material used (i.e., concrete or timber). The proposed novel bond–slip model can reflect all stages of the bond response in one single function, the three stages being an elastic stage, a softening stage, and a friction stage. The proposed function is shown in Equation (12a,b), where $\tau_{bmax}$ is the peak bond stress, $\tau_{bf}$ is the residual stress, $s_{t}$ is the slip corresponding to the midpoint of the transition between the maximum and the residual stresses, and a and b are calibrated parameters. Figure 27 shows a comparison of the proposed model with experimental pullout tests on ribbed steel bars of varying diameter. Figure 28 shows a comparison between experimental results from [76] and model results for GFRP bars at different temperatures. Furthermore, Figure 29 compares experimental results (dashed line) from [129] and model results (Solid line) for sand-coated and smooth GFRP bars.

(12a) $$\frac{\tau_{b}(s)}{\tau_{bmax}}=(1-e^{-b\cdot s})\cdot \frac{\alpha +e^{-a\cdot (s-s_{t})}}{1+e^{-a\cdot (s-s_{t})}}$$

(12b) $$\alpha =\frac{\tau_{bf}}{\tau_{bmax}}$$

## 4. Calibration and Comparison of Theoretical Models

**Figure 30:**
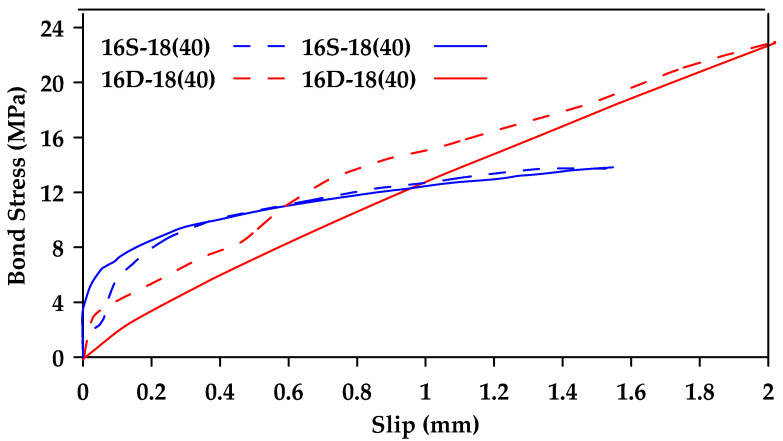
Calibrated modified BPE model and experimental results for bond–slip relationship for 16 mm GFRP bars of different rib depth. Data from [130].

**Figure 31:**
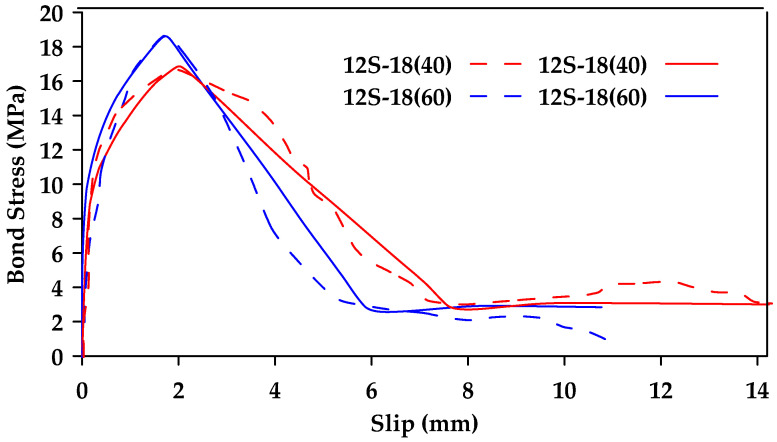
Calibrated modified BPE model and experimental results for bond–slip relationship for 12 mm GFRP bars embedded in concretes of different strengths. Data from [130].

Zhang et al. [130] investigated the influence of rib depths and spacing on the bond behavior between GFRP rebar and concrete by performing pullout tests [130]. A significant increase in bond strength (about 55%) was observed as a result of increasing the rib depth from 0.5 to 1.5 mm. On the other hand, rib spacing had a minimal effect on bond strength. Furthermore, the test results were used to calibrate the modified BPE model expressions for GFRP bars with different rib depths and spacing. The results indicate proximity between the calibrated model results and the experimental results (Figure 30 and Figure 31). In the figure, the designation format followed is $d_{b}x-r({f^{\prime }}_{c})$, where $d_{b}$ is the bar diameter, x represents the rib depth (s for shallow and d for deep), r is the rib spacing, and ${f^{\prime }}_{c}$ is the concrete strength.

**Figure 32:**
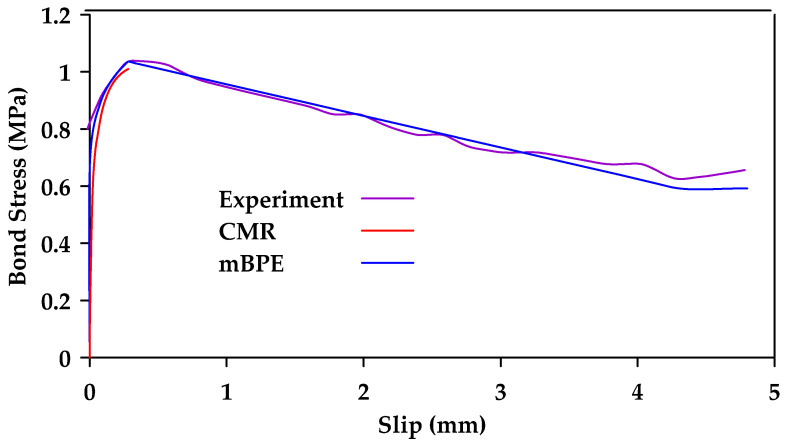
Calibration of mBPE and CMR for smooth GFRP bars. Data from [124].

**Figure 33:**
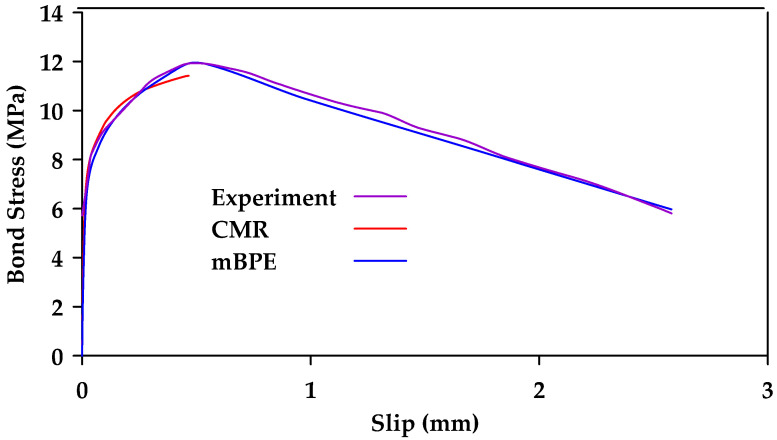
Calibration of mBPE and CMR for ribbed GFRP bars. Data from [124].

**Figure 34:**
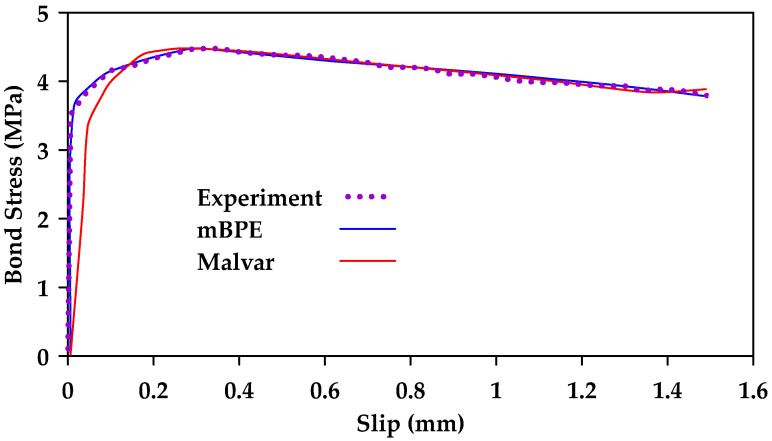
Calibration of mBPE and CMR for twisted polyethylene GFRP bars. Data from [124].

Cosenza et al. [124] calibrated the modified BPE model, the CMR model, and the Malvar Model against experimental results. The study included seven different types of bar surfaces. Table 1 shows the mean values of the model parameters for the different bar surfaces for the mBPE and CMR models. Furthermore, Figure 32 and Figure 33 show the calibrated models against experimental results for smooth and ribbed bar surfaces, respectively. The authors concluded that the modified BPE model is suitable for reproducing the entire bond slip curve and shows a good agreement with the experimental data, even within the first branch. On the other hand, the CMR model led to the best simulations of the ascending branch. The Malvar model was found to be able to reproduce the entire constitutive curve, but is less reliable in modelling the ascending branch than the modified BPE and the CMR models. It is noted that the study only shows a comparison between the Malvar model and the modified BPE model for polyethylene and carbon fiber-reinforced polymer bars. It is implied that additional comparisons were made for GFRP bars with similar results. However, these tests and comparisons are not shown explicitly in the paper. Figure 34 shows instead the comparison between the modified BPE model and the Malvar model for twisted polyethylene bars.

**Figure 35:**
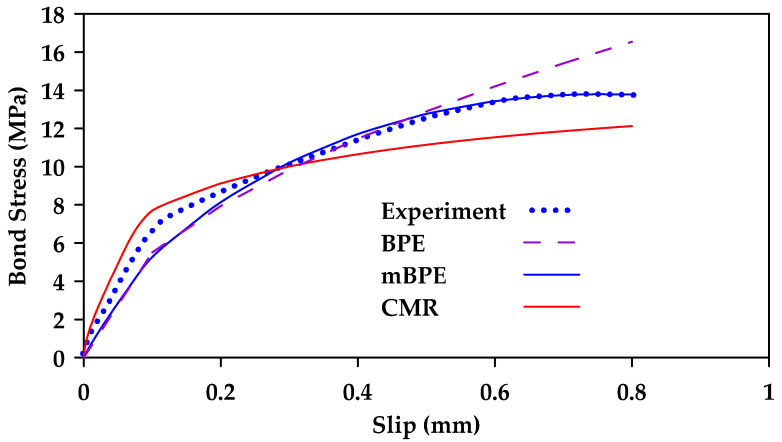
Calibration of theoretical bond slip models (BPE, mBPE, and CMR) for ribbed bars. Data from [131].

**Figure 36:**
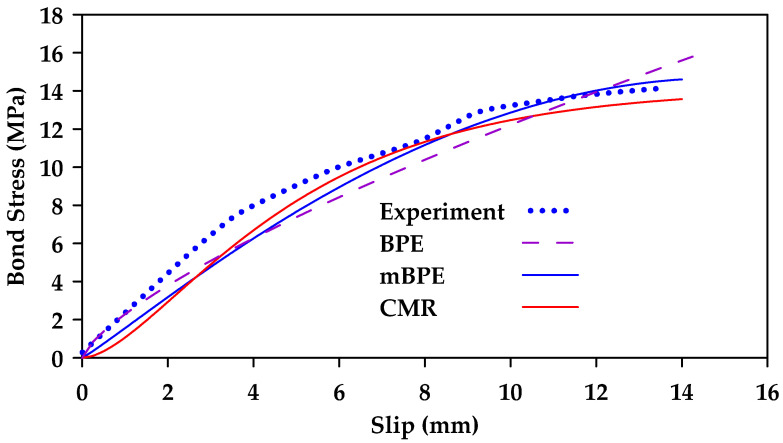
Calibration of theoretical bond slip models (BPE, mBPE, and CMR) for helically wrapped sand-coated bars. Data from [131].

Aiello et al. [131] calibrated the ascending branch of various theoretical models for different bar surfaces (ribbed and helically wrapped sanded rebars) [131]. The models considered were the BPE model, the CMR model, and the modified BPE model. Figure 35 and Figure 36 show a comparison between the results obtained for ribbed and helically wrapped GFRP bars. The modified BPE model produces the best results for ribbed bars. On the other hand, both the modified BPE model and the CMR model produce acceptable results for helically wrapped bars.

**Figure 37:**
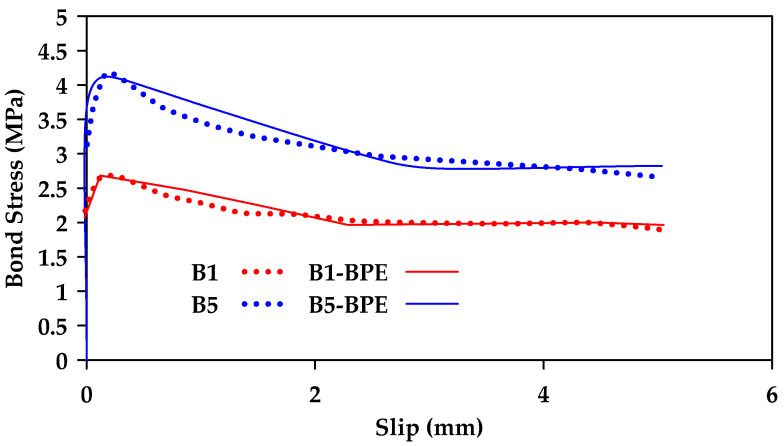
Comparison between BPE model and experimental results for rough 12 mm GFRP bars embedded in high (B5) and medium strength concrete (B1). Data from [123].

In another study, Rossetti et al. [123] investigated the reliability of the BPE model in simulating the bond behavior of 12 mm GFRP bars with both rough and smooth surfaces. The difference between the specimens was in the concrete strength used. Figure 37 shows the proximity between the model and the experimental results for the rough bars. The calibration parameters are shown in Table 2. It is noted that values used for S1 and S2 are very close, which shows that the constant stage of the BPE model (see above) is negligible. Additionally, the descending branch is not captured as reliably as the ascending branch. These two observations highlight that the modified BPE model is more suitable for modelling the bond of GFRP bars than the BPE model.

## 5. Expressions for Bond Strength

In addition to the aforementioned models, some studies and design codes have proposed expressions to estimate the bond strength between GFRP bars and concrete. While these models do not show the full bond behavior, they do account for different parameters that influence bond strength. For example, Okelo and Yuan [132] proposed an expression for calculating the average bond strength for a straight FRP bar embedded in normal-strength concrete as a function of the concrete strength and bar diameter [132] (Equation (13)). In Equation (13), $u_{f}$ is the average bond strength, ${f^{\prime }}_{c}$ is the concrete strength, and $d_{b}$ is the bar diameter. This equation was calibrated based on the results obtained from 151 pullout tests performed on different types of FRP rebars (AFRP, CFRP, and GFRP). Lee et al. [113] also proposed an expression to estimate bond strength of FRP reinforced members as a function of concrete strength only (Equation (14)) [133].

(13) $$u_{f}=14.7\frac{\sqrt{{f^{\prime }}_{c}}}{d_{b}}$$

(14) $$u_{f}=3.3{f^{\prime }}_{c}^{0.3}$$

In its 2006 Report, ACI committee 440 endorsed the expression developed in [134] to estimate the bond of FRP bars as shown in Equation (15). In Equation (15), $L_{d}$ is the development length and c is the smallest value between the side cover, bottom cover, and half the clear spacing between bars. The equation is applicable to glass, aramid, and carbon FRP bars and accounts for the bar diameter, embedment length, and concrete strength. However, the equation does not account for the presence of confinement, fiber type, or the bar surface.

(15) $$\frac{u_{f}}{0.083\sqrt{{f^{\prime }}_{c}}}=4.0+0.3\frac{C}{d_{b}}+100\frac{d_{b}}{L_{d}}\leq 9.0$$

One expression that is calibrated for GFRP bars and considers the bar surface in addition to the bar diameter, embedment length, and concrete strength was proposed in [38]. The expression makes use of the term $R_{r}$ which represents the ratio of the bearing area to the shearing area of the ribs of the GFRP bar as shown in Equation (16).

(16) $$u_{f}=\sqrt{{f^{\prime }}_{c}}(0.1337+0.1539\frac{C}{d_{b}}+2.673+5.22R_{r})$$

In summary, several models have been proposed to simulate both the bond strength of GFRP bars to concrete and the full behavior. Table 3 provides a summary of both types of models and their required parameters.

## 6. Discussion

This section provides a discussion of the factors that affect bond slip and the numerical models that simulate it.

### 6.1. Factor Affecting Bond

Based on the presented review, several conclusions can be made about the bond behavior of GFRP bars embedded in concrete. It is observed that bond behavior is influenced by several factors: bar diameter, bar surface, concrete strength, and embedment length. These factors are discussed in detail in the sections below. Note that in addition to the discussion of factors, tables are provided that summarize the different trends as observed in the experimental studies discussed above. The spread in some of the results highlighted can be primarily attributed to the variability in surface treatments and textures. For example, different studies might refer to their GFRP bars as ribbed bars. However, these bars might have different properties (rib depth and spacing), which will affect the outcome. Similar observations can be made or sand-coated bars, where the roughness of the sand coating can vary between experiments.

#### 6.1.1. Bar Diameter

**Figure 38:**
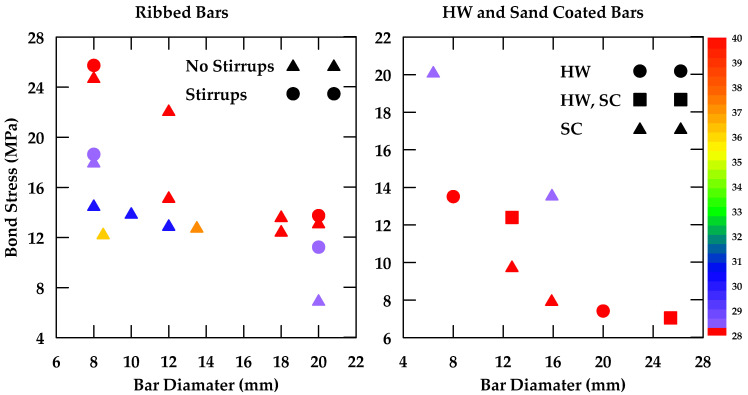
The effect of bar diameter on the bond slip of GFRP bars of different surfaces embedded in concrete of different strengths.

The bar diameters studied ranged from 8 mm to 20 mm. In general, the bond strength of GFRP bars decreases with an increase in bar diameter. This effect is observed regardless of the bar surface under consideration. This relationship could be attributed to shear lag effects and Poisson’s ratio. As a bar is subjected to axial stress, Poisson’s ratio leads to a reduction in the original bar diameter, which diminishes the contact area and friction between the strand and the surrounding concrete, leading to a reduction in the bond strength. As the bar diameter increases, the reduction associated with Poisson’s effect also increases, leading to a lower bond strength. Furthermore, the shear lag effect, which can lead to a reduction in bond strength and is more significant in larger bar diameters, also contributes to the lower bond strength observed in larger bar diameters [135]. This finding was also confirmed in [136], where it was experimentally and numerically determined that significant shear lag effects occur in fiber-reinforced polymers, with larger shear lag effects observed the lower the shear stiffness (i.e., larger bar diameters lead to larger shear lag effects). Bi et al. [137] also attributed the lower bond strength when the bar diameter increased to the higher quantity of bleeding water trapped beneath the bar, resulting in greater voids [137]. This reduces the contact surface between the bar and concrete, and consequently, bond strength decreases. It was also observed that in some cases, the bar diameter can alter the failure mode, with larger bar diameters being more prone to pullout failure since the effective surface area per unit force for larger bar diameters is lower, which limits the radial tensile stresses transmitted to the concrete and reduces the possibility of splitting. Table 4 summarizes the effect of bar diameter on bond strength from selected studies. Furthermore, Figure 38 is a graphical representation of this effect on GFRP bars of different surfaces embedded in concrete of different strengths.

#### 6.1.2. Concrete Strength

Concrete strength can also impact the bond strength and the associated failure mode due to increased mechanical interlocking and friction between the concrete and the bar. The range of concrete strength studied varied from 15 MPa to 62 MPa. Two main observations could be made regarding the impact of concrete strength. First, if the specimen uses weak concrete (<30 MPa), then increasing the concrete strength can lead to a significant increase in bond strength as the concrete is better able to resist local crushing, splitting, and shear at the interface. However, if the specimen uses a relatively stronger concrete (>30 MPa), the increase in bond strength with an increase in concrete strength is significantly less pronounced as the failure is more controlled by the bar–concrete interface rather than the concrete’s crushing or splitting resistance. In addition, if the specimen is prone to splitting failure, then increasing the concrete strength could increase the bond strength and alter the failure mode to pullout failure, since stronger concrete can resist higher radial (splitting) forces. The impact of concrete strength on bond behavior is also highly dependent on the bar surface under consideration. For example, the bond of smooth GFRP bars relies on adhesion for bond and is not affected by concrete strength, while other surfaces rely on mechanical interlock, which is dependent on concrete strength. Table 5 summarizes the effect of concrete strength on bond strength from selected studies. In addition to the studies conducted on normal concrete, several studies have concluded that the use of high-performance concrete, such as geopolymer concrete, can lead to improved bond behavior with GFRP bars due to their improved mechanical properties and dense microstructure. Furthermore, the addition of steel fibers to concrete can also affect bond strength as they can prevent propagation of cracks and increase ductility, which helps maintain the integrity of the bond interface.

#### 6.1.3. Confinement

**Figure 39:**
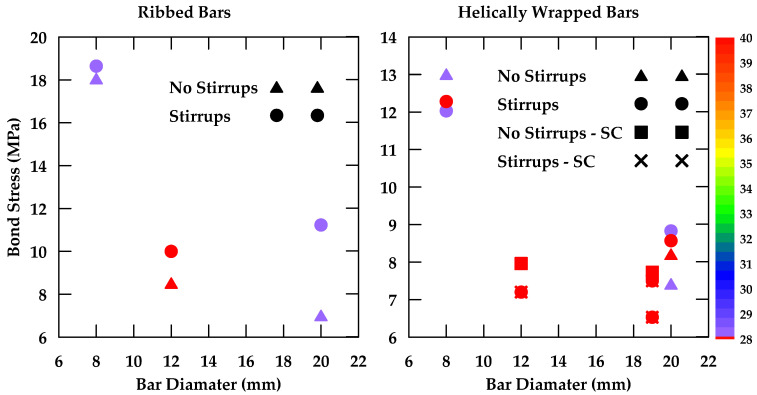
The effect of confinement on the bond slip of GFRP bars of different surfaces and diameters embedded in concrete of different strengths.

Table 6 and Figure 39 summarize the effect of confinement on bond strength. This effect can vary depending on the bar surface and concrete strength. For example, confinement has a minimal effect on bond strength in the case of helically wrapped bars. This is because the bond mechanism for helically wrapped GFRP bars relies primarily on the friction and adhesion between the bar surface and the concrete, rather than a strong mechanical interlock as is the case with ribbed GFRP bars. Conversely, confinement leads to a significant improvement in the bond behavior of ribbed GFRP bars, particularly larger diameters, due to increased mechanical interlock. The use of confining stirrups could also shift the failure mode from concrete splitting to a more ductile bar pullout failure, particularly in bars embedded in weaker concrete, as the addition of confining stirrups provides additional resistance to the splitting forces and can delay cracking. On the other hand, the addition of confining stirrups has a reduced effect on the bond strength of GFRP bars embedded in stronger concretes, as these specimens were likely to fail due to pullout rather than concrete splitting, and therefore, the addition of stirrups will not alter the failure mode.

#### 6.1.4. Bar Surface

Table 7 shows that the bar surface has arguably the most prominent effect on bond strength, as bars with helically wrapped and smooth surfaces exhibit significantly lower bond strength than ribbed bars due to the reduced mechanical interlock. Furthermore, bars with a smaller diameter are affected by the bar surface more than bars with a larger diameter. This is because the surface area to cross-sectional area ratio is higher for smaller diameters, which means that a larger proportion of the bar’s circumference is in contact with the surrounding concrete. Additionally, sand coating can increase the bond strength of helically wrapped bars as it creates a larger contact surface between the bars and the concrete and therefore increases friction and enhances mechanical interlock. Additionally, sand coating can improve chemical adhesion as cement paste can anchor better to the irregular sand particles compared to smooth surfaces. The performance of ribbed bars is improved with the use of deeper ribs, as deeper ribs improve the grip and lead to stronger mechanical interlock. However, if the rib depth is excessive, the opposite effect may occur as the failure may change from pullout failure to splitting failure, and bond strength will decrease.

#### 6.1.5. Embedment Length

In general, bond strength decreases when the embedment length increases. This effect, which is more prominent for larger bar diameters, may be attributed to the nonlinear distribution of bond stress along the bar [11]. As the embedment length increases, the bond stress decreases as it is distributed over a longer length [138]. This effect is more prominent for larger diameters as the non-uniformity of the bond stress distribution is even greater. In addition, an increase in embedment length can lead to a lower initial stiffness in the bond behavior, since it takes longer for the full embedment length to engage as the embedment length increases. However, despite the reduction in bond strength, an increased embedment length can lead to a higher overall slip due to the longer length available to develop deformation. Table 8 summarizes the effect of embedment length on bond strength from selected studies.

#### 6.1.6. Aggressive Environment and Temperature

A significant number of studies have been conducted to study the durability of the bond of GFRP bars. These studies comprise subjecting GFRP bars embedded in concrete to aggressive environments, such as alkaline solutions, acid solutions, seawater, and direct sunlight. The findings from most of these studies have concluded that the bond of GFRP bars is unaffected by these aggressive agents, regardless of exposure duration. This is because the bond is governed by mechanical interlock and friction, which are affected by environmental agents, unlike chemical adhesion. Additionally, both the glass and resin used in the manufacture of GFRP bars have good resistance to aggressive agents. On the other hand, research on the effect of temperature on the bond of GFRP bars has concluded that the bond significantly deteriorates with increased temperature. The decline in temperature starts at around 80 °C, and a reduction of about 80−90% in bond strength is observed at a temperature of 300 °C. The reduction in bond strength with temperature is like that observed in steel bars. This decline in bond occurs because at elevated temperatures, the resin in the GFRP bars begins to soften and decompose, which reduces the integrity of the bar surface.

### 6.2. Numerical Models

The typical bond stress–slip plot for GFRP bars is composed of an ascending branch, corresponding to the chemical adhesion and bearing force between the bar and concrete a, a descending branch, corresponding to cracking and crushing of concrete, as well as a stage of constant stress due to friction. Different models have been proposed to simulate the bond stress–slip relationships of GFRP bars embedded in concrete. These models include the BPE model, the modified BPE model, and the CMR model. The models have been calibrated against experimental results with the modified BPE model producing the best approximation of the overall bond stress–slip relationships of GFRP bars, while the CMR model produces a more accurate approximation of the ascending branch of the bond stress–slip relationships. Other researchers have proposed more recent models that have also produced a certain degree of accuracy [40,126,128]. However, none of these models account explicitly for the effect of different factors (bar diameters, concrete strength…) on the bond. Instead, the models require calibration with available experimental results. Therefore, these bond slip models are useful for running calibration simulations and limited parametric studies. There are several reasons that limit the ability of numerical models to explicitly account for the different factors that affect bond slip. First, the contact friction at the bar–concrete interface is difficult to model due to its nonlinear and variable nature and the fact that it depends on complex interactions, such as micro-cracking, roughness evolution, and local stress states. Additionally, the development and progression of micro-damage in the concrete is sensitive to concentrations of local stress, making it challenging for simplified models to capture. Furthermore, adhesion modeling challenges arise from the lack of well-defined physical laws describing chemical bonding at the interface (particularly for materials like GFRP), and from the difficulty in measuring adhesion contributions separately from friction and mechanical interlock experimentally. However, in order to allow civil engineers and researchers to fully incorporate the effect of bond slip in their designs and numerical simulations, it is still necessary that the bond slip models account implicitly for the various factors that affect bond behavior in a manner such that users can estimate the behavior of a specific GFRP bar without the need for extensive pullout tests despite the difficulty and challenges facing such a task.

## 7. Conclusions

This study presented an overview of experimental and analytical investigations on the bond slip of GFRP bars in concrete. The results of experimental tests were discussed in detail. Furthermore, a summary of available analytical models and expressions that estimate bond behavior and bond strength were presented. The following conclusions can be made:

- Bond slip is influenced by several factors: bar diameter, bar surface, concrete strength, and embedment length
- In general, the bond strength of GFRP bars decreases with an increase in bar diameter.
- The effect of concrete strength on the bond behavior can vary depending on the bar type and failure mode. In general, bond strength increases with an increase in concrete strength.
- The bar surface also has a prominent effect on the bond strength and failure mode, with ribbed bars having a higher bond strength and failing due to concrete splitting, while smooth bars typically fail due to pullout of the bar.
- Confinement can increase the bond strength of GFRP bars.
- It is important to note that the majority of experimental tests in the literature are biased towards standard pullout tests, which do not fully represent the complex stress conditions occurring in real structural elements.
- Another limitation of the present review stems from the inherent variability in test conditions reported across different studies. Although pullout tests follow standard procedures, significant differences in key experimental parameters can affect the comparability of results and the generalizability of conclusions. For example, surface treatment (e.g., ribbed, sand-coated, helically wrapped), and material properties (e.g., steel vs. GFRP) are not always consistently reported or standardized, introducing variability that affects bond strength and failure mode. Additionally, the wide range of concrete compressive strengths, mix designs, aggregate types, and curing conditions makes it difficult to directly compare bond strength or bond–slip behavior across studies without normalizing or adjusting for these factors.
- Several analytical models have been proposed to simulate the bond behavior between GFRP bars and concrete. Both traditional and more recent models require calibration with experimental results but do not explicitly account for the effect of different factors (bar diameters, concrete strength) on the bond.
- This review helps improve guidance for the selection and modeling of GFRP reinforcement in structural design. The summarized data can support more reliable input parameters for finite element modeling, and the discussion of code developments informs ongoing efforts to standardize design methodologies for GFRP-reinforced concrete elements.
- Future research must be focused on developing expressions to model bond behavior that implicitly account for different parameters without the need for calibration. This will allow researchers and engineers to accurately account for bond slip in GFRP bars across different types of bars and bar diameters and is thus an essential step for the commercialization of GFRP-reinforced concrete.
- Another focus for researchers should be to establish uniform guidelines for material composition, manufacturing processes, and testing procedures in order to unify production standards for Glass Fiber-Reinforced Polymer (GFRP) bars across manufacturers. This step is not only necessary for ensuring consistency, reliability, and safety in engineering projects but also for modeling and accounting for bond slip, which is highly dependent on the characteristics of the bars (i.e., surface, ribs, manufacturing process) as demonstrated in this review.

## Figures and Tables

**Table 1:** Mean values of the model parameters for the different bar surfaces. The number in brackets refers to the number of samples considered.

Bar Surface	Modified BPE			CMR	
	α	ρ	$\tau_{3}$	β	$S_{r}$
Smooth (10)	0.145	1.87	0.99	0.314	0.11
Grain covered (5)	0.067	3.11	3.17	0.138	0.07
Sandblasted (8)	0.251	2.63	1.38	0.559	0.41
Twisted (3)	0.175	4.15	3.68	0.593	0.12
Ribbed (27)	0.283	14.88	7.79	0.575	0.45
Indented and braided (12)	0.177	12.8	6.26	0.473	0.78
Braided and sanded (2)	0.069	0.95	7.13	0.025	0.08

**Table 2:** BPE model parameters.

Bar Designation	$\tau_{1}$	$S_{1}$	$S_{2}$	$S_{3}$	α	β
B1	2.86	0.25	0.25	3.47	0.16	0.77
B5	4.17	0.19	0.2	2.64	0.12	0.67

**Table 3:** Model summary.

Model	Type	Main Parameters	Equation
Malvar (1994) [120]	Behavior	Concrete tensile strength, peak bond stress, slip at peak bond stress, the confining axisymmetric radial pressure	Equation (1)
BPE model	Behavior	peak bond stress, slip at peak bond stress	Equation (2a–c)
MBPE model	Behavior	peak bond stress, slip at peak bond stress	Equation (3a–d)
CMR model	Behavior-Ascending Branch	peak bond stress, slip at peak bond stress	Equation (4a–c)
Tighiouart et al. (1998) [53]	Behavior-Ascending Branch	peak bond stress, slip at peak bond stress	Equation (5)
Gooranorimi et al. (2017) [40]	Behavior-Ascending Branch	peak bond stress, slip at peak bond stress	Equations (6) and (7)
Rezazadeh et al. (2017) [126]	Behavior	peak bond stress, slip at peak bond stress, elastic bond stiffness	Equations (7)–(9a–c)
Biscaia and Carmo (2023) [128]	Behavior	peak bond stress, residual stress, slip corresponding to the midpoint between the maximum and the residual stress	Equation (11a,b)
Okelo and Yuan (2005) [132]	Peak Bond Strength	Concrete compressive strength, bar diameter	Equation (12a,b)
Lee al. (2008) [133]	Peak Bond Strength	Concrete compressive strength	Equation (13)
Wambeke and Shield (2006) [134]	Peak Bond Strength	Concrete compressive strength, bar diameter, embedment length, concrete cover	Equation (14)
Rather et al. (2024) [38]	Peak Bond Strength	Concrete compressive strength, bar diameter, concrete cover, ratio of the bearing area to the shearing area of the ribs	Equation (15)

**Table 4:** Effect of bar diameter on bond strength.

Bar Diameter	Concrete Strength	Bar Surface	Confinement	Embedment Length	Bond Strength	Citation
**8**	**28**	**HW**	**N**	5 d	**13.51**	Gao et al. (2019) [37]
**20**	**28**	**HW**	**N**	5 d	**7.42**	
8	28	Ribbed	N	5 d	18.06	
20	28	Ribbed	N	5 d	7.01	
**8**	**28**	**Ribbed**	**Y**	5 d	**18.64**	
**20**	**28**	**Ribbed**	**Y**	5 d	**11.23**	
8	40	Ribbed	N	5 d	24.8	
20	40	Ribbed	N	5 d	13.2	
**8**	**40**	**Ribbed**	**Y**	5 d	**25.74**	
**20**	**40**	**Ribbed**	**Y**	5 d	**13.75**	
6.4	38	SC	N	5 d	20.15	Ahmed et al. (2008) [41]
15.9	38	SC	N	5 d	13.61	
**8.5**	**36**	**Ribbed**	**N**	4 d	**12.32**	Achillides and Pilakoutas (2004) [42]
**13.5**	**37**	**Ribbed**	**N**	4 d	**12.85**	
12.7	31	HW, SC	N	20 d	12.4	Benmokrane and Tighiouart (1996) [44]
25.4	31	HW, SC	N	20 d	7.05	
**12.7**	**40**	**SC**	**N**	**5 d**	**9.8**	Tastani and Pantazopoulou (2006) [32]
**15.88**	**40**	**SC**	**N**	**5 d**	**8.0**	
12	40	Shallow ribbed	N	5 d	15.23	Chen at al. (2023) [45]
18	40	Shallow Ribbed	N	5 d	12.53	
**12**	**40**	**Deep Ribbed**	**N**	5 d	**22.16**	
**18**	**40**	**Deep Ribbed**	**N**	5 d	**13.7**	
8	30	Ribbed	N	5 d	14.58	Hao et al. (2009) [46]
10	30	Ribbed	N	5 d	13.96	
12	30	Ribbed	N	5 d	12.99	

**Table 5:** Effect of concrete strength on bond strength.

Concrete Strength	Bar Diameter	Bar Surface	Confinement	Embedment Length	Bond Strength	Citation
**28**	**8**	**Ribbed**	**N**	5 d	**18.06**	Gao et al. (2019) [37]
**40**	**8**	**Ribbed**	**N**	5 d	**24.8**	
28	20	Ribbed	N	5 d	7.01	
40	20	Ribbed	N	5 d	13.2	
**28**	**8**	**Smooth**	**N**	5 d	**13.51**	
**40**	**8**	**Smooth**	**N**	5 d	**12.16**	
28	20	Smooth	N	5 d	7.42	
40	20	Smooth	N	5 d	8.21	
**32**	**12**	**Ribbed**	**N**	**5 d**	**8.52**	Rather et al. (2024) [38]
**60**	**12**	**Ribbed**	**N**	**5 d**	**12.803**	
24	8	Ribbed	N	5 d	8.4	Veljkovic et al. (2017) [43]
39	8	Ribbed	N	5 d	9.5	
56	8	Ribbed	N	5 d	11.9	
62	8	Ribbed	N	5 d	15.5	
**15**	**13.5**	**Ribbed**	**N**	**4 d**	**3.1**	Achillides and Pilakoutas (2004) [42]
**37**	**13.5**	**Ribbed**	**N**	**4 d**	**12.8**	
**45**	**13.5**	**Ribbed**	**N**	**4 d**	**11.9**	

**Table 6:** Effect of confinement on bond strength.

Confinement	Bar Diameter	Concrete Strength	Bar Surface	Embedment Length	Bond Strength	Citation
**N**	**8**	**28**	HW	5 d	**13.01**	Gao et al. (2019) [37]
**Y**	**8**	**28**	HW	5 d	**12.03**	
N	20	28	HW	5 d	7.42	
Y	20	28	HW	5 d	8.83	
**N**	**8**	**40**	HW	5 d	**12.16**	
**Y**	**8**	**40**	HW	5 d	**12.28**	
N	20	40	HW	5 d	8.21	
Y	20	40	HW	5 d	8.57	
**N**	**8**	**28**	**Ribbed**	5 d	**18.06**	
**Y**	**8**	**28**	**Ribbed**	5 d	**18.64**	
N	20	28	Ribbed	5 d	7.01	
Y	20	28	Ribbed	5 d	11.23	
**N**	**12**	**32**	**Ribbed**	5 d	**8.52**	Rather et al. (2024) [38]
**Y**	**12**	**32**	**Ribbed**	5 d	**10**	
N	12	32	Ribbed	10 d	7.86	
Y	12	32	Ribbed	10 d	9.17	
**N**	**12**	**40**	**HW, SC**	**5 d**	**7.96**	Tastani and Pantazopoulou (2006) [32]
**Y**	**12**	**40**	**HW, SC**	**5 d**	**7.2**	
N	19	40	HW, SC	5 d	7.63	
Y	19	40	HW, SC	5 d	7.5	
**N**	**19**	**40**	**HW, SC**	**5 d**	**7.73**	
**Y**	**19**	**40**	**HW, SC**	**5 d**	**6.53**	

**Table 7:** Effect of bar surface on bond strength.

Bar Surface	Bar Diameter	Concrete Strength	Confinement	Embedment Length	Bond Strength	Citation
**HW**	**8**	**28**	**N**	5 d	**13.51**	Gao et al. (2019) [37]
**HW, SC**	**8**	**28**	**N**	5 d	**16.19**	
**Ribbed**	**8**	**28**	**N**	5 d	**18.06**	
HW	20	28	N	5 d	7.42	
HW, SC	20	28	N	5 d	7.11	
Ribbed	20	28	N	5 d	7.01	
**HW**	**8**	**40**	**N**	5 d	**12.6**	
**Ribbed**	**8**	**40**	**N**	5 d	**24.8**	
Smooth	12	40	N	5 d	4.41	Chen et al. (2023) [45]
Shallow Rib	12	40	N	5 d	15.23	
Deep Rib	12	40	N	5 d	22.16	
**Smooth**	**18**	**40**	**N**	**5 d**	**4.7**	
**Shallow Rib**	**18**	**40**	**N**	**5 d**	**12.53**	
**Deep Rib**	**18**	**40**	**N**	**5 d**	**13.7**	

**Table 8:** Effect of embedment length on bond strength.

Embedment Length	Bar Diameter	Concrete Strength	Bar surface	Confinement	Bond Strength	Citation
5 d	12	32	Ribbed	N	8.52	Rather et al. (2024) [38]
10 d	12	32	Ribbed	N	7.86	
5 d	**12**	**32**	**Ribbed**	**Y**	**10**	
10 d	**12**	**32**	**Ribbed**	**Y**	**9.17**	
2 d	13.5	37 (35)	Ribbed	N	13.65	Achillides and Pilakoutas (2004) [42]
4 d	13.5	37 (35)	Ribbed	N	12.85	
**2 d**	**13.5**	**45**	**Ribbed**	**N**	**11.7**	
**4 d**	**13.5**	**45**	**Ribbed**	**N**	**10**	
**6 d**	**13.5**	**45**	**Ribbed**	**N**	**11.9**	
**8 d**	**13.5**	**45**	**Ribbed**	**N**	**8.9**	
**10 d**	**13.5**	**45**	**Ribbed**	**N**	**9.1**	
2 d	13.5	15	Ribbed	N	2.8	
4 d	13.5	15	Ribbed	N	3.1	
6 d	13.5	15	Ribbed	N	1.9	
8 d	13.5	15	Ribbed	N	2.5	
10 d	13.5	15	Ribbed	N	2.6	

## Data Availability

No new data were created or analyzed in this study. Data sharing is not applicable to this article.

## Abbreviations

The following abbreviations are used in this manuscript:

GFRP	Glass Fiber Reinforced Polymer
BPE	Bertero, Popov, and Eligehausen Bond Model
mBPE	Modified BPE Model
CMR	Cosenza-Manfredi-Realfonzo Bond Model
